# Aggregation of Graphene Flakes Under Electric Field: A Molecular Simulation Study

**DOI:** 10.1021/acsomega.5c11004

**Published:** 2026-02-03

**Authors:** Jiang Wang, Zaigui Yang, Yiping Shi, Guangxiang Wei, Zhiling Li

**Affiliations:** College of Science, 419887 Guizhou Institute of Technology, Boshi Road, Huaxi District, Guiyang, Guizhou 550025, China

## Abstract

Graphene flakes, as two-dimensional materials, can self-assemble under certain conditions and have wide-ranging applications in industries from electronics to biomedicine due to their exceptional mechanical, thermal, and electrical properties. Recent studies indicate that single graphene flakes can be aligned by an external electric field (EF) in polar solvents like water. However, how their self-assembly behavior is influenced by the EF remains unclear. In this work, we use molecular dynamics (MD) simulations to explore the self-assembly of graphene flakes with different shapes and sizes under various EF conditions: static EF (SEF), alternating EF (AEF), and circularly polarized EF (CPEF). Our results reveal that different EF conditions significantly impact the number of pairwise bindings between flakes and the average size of the aggregates. In the absence of an EF, graphene flakes tend to form globular, round-like structures. When an EF is applied, particularly under SEF, the aggregates adopt a stretched configuration aligned with the field’s direction, while AEF has a weaker alignment ability than SEF. Under CPEF, elongated aggregates rotate, following the field with a characteristic lag angle. Furthermore, while aggregates can explore more configurations under CPEF, the two-dimensional free energy landscape indicates that the stretched state is the most stable. This work deepens our understanding of how EFs influence the self-assembly of graphene flakes, potentially guiding future engineering applications for controlling the aggregation of graphene and other discotic molecules.

## Graphical abstract

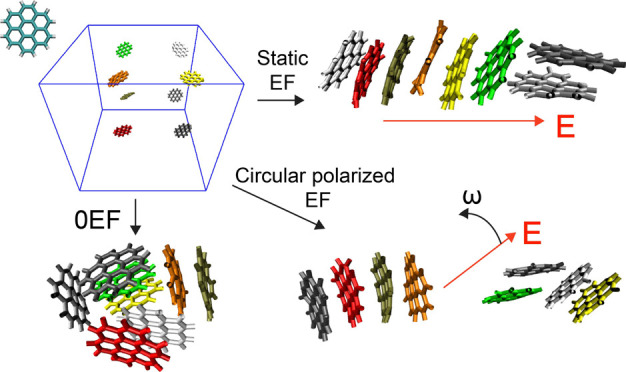

## 1 Introduction

Graphene is a well-known two-dimensional material composed of carbon atoms. Each atom is connected to three adjacent atoms via σ-bonds and delocalized π-bonds, forming a single-layer honeycomb structure.^1^ Since its discovery, graphene has demonstrated a range of exceptional properties, including high tensile strength, thermal conductivity, large surface area, excellent optical transmittance, and high electrical conductivity.^2^ These properties have attracted significant attention from both academic research and industrial applications, such as transistors, transparent displays, coatings, healthcare and medicine, photoelectronics, friction-reduction and antiwear manufacturing, as well as electrical and tissue engineering.^1,3–5^

When a graphene flake serves as a rigid aromatic core and is grafted with “hairy” side chains, it forms a typical discotic liquid crystal (DLC).^6,7^ DLCs have numerous industrial applications due to their ability to self-assemble into one-dimensional (1D) rod-like structures.^8^ The delocalized π electrons can transfer along these assembled 1D rods, granting them exceptional electro-optical properties. This has led to their widespread use in organic electronic and semiconductor devices, such as organic light-emitting diodes (OLEDs), field-effect transistors, and discotic-based photovoltaic solar cells.^6,9^

The driving forces for the self-assembly of graphene and DLCs in solvents are π–π interactions,^10,11^ π-σ interactions,^12^ and hydrophobic interactions.^13^ Under the influence of these interactions, graphene flakes tend to attract and stack together, forming aggregates with specific configurations.^3–5^ The resulting aggregation profile depends on the environmental conditions, such as the solvent type and the shape of the constituent building blocks.^14,15^

The use of an external EF to assist chemical reactions has become a popular approach for effectively controlling chemical processes. Numerous experimental studies have demonstrated that a specific external EF can align graphene flakes in a particular direction and influence their aggregation process.^16–24^ Applying SEF and AEF to graphene can enhance its performance in various applications, such as improving the anticorrosive reinforcement of epoxy coatings,^23^ enhancing the multifunctional properties of epoxy nanocomposites,^16^ and increasing anisotropic electrical properties.^3^

Molecular dynamics simulations have become a useful tool for understanding chemical processes, providing access to atomic-level details. MD has been applied to study various graphene properties,^2,25–31^ the alignment of graphene under electric fields,^17,32,33^ and the self-assembly of graphene and related discotic molecules.^34–37^

One mechanism by which an external EF can control the alignment and orientation of graphene or other carbon nanoparticles, such as carbon nanofibers^38,39^ and carbon nanotubes,^40–42^ is through the induction of polarization and a dipole moment in these particles. Conversely, our recent work shows that, in addition to the induced dipole moment on carbon nanoparticles, the polar solvent effect can also influence the configuration and alignment of flexible polymers or rigid graphenes, even when no dipole moment is induced on the molecules themselves.^33,43,44^

Under an external EF, the dipole moments of a polar solvent such as water become oriented, leading to the formation of a directional hydrogen-bond network and 1D water nanowires.^43–50^ These directional 1D water structures subsequently influence the orientation of solute molecules, aligning them in a way that minimizes disruption to the ordered hydrogen-bond network.^33,43,44^ In our recent work, we demonstrate that different types of EFs could exert specific influence on the alignment and rotational behavior of a single graphene flake.^43^

In this paper, we utilize MD to explore how different types of external electric fields-SEF, AEF, and CPEF-as well as the shape of the graphene flakes, impact the aggregation of graphene in water. We assume an electrostatic condition in which the external EF does not induce an additional dipole moment or polarization in the graphene flakes. This approach allows for a more specific understanding of the polar solvent effect in modulating the aggregation process.

This study advances our understanding of how EF modulate the self-assembly of graphene and other discotic molecules, while also offering valuable insights for industrial applications in which EFs are employed to control molecular aggregation.

## 2 Methods

### 2.1 Molecular Dynamics Simulation

We study two graphene flakes with different shapes in this work, **G2**–**1** (C_24_H_12_) and **G2**–**2** (C_52_H_20_), as shown in Figure 1. **G2**–**1** has a symmetrical shape with two layers of aromatic rings and an aspect ratio of 1. **G2**–**2** is similar to **G2**–**1** in its number of aromatic ring layers but has an aspect ratio of approximately 2.

**Figure 1:**
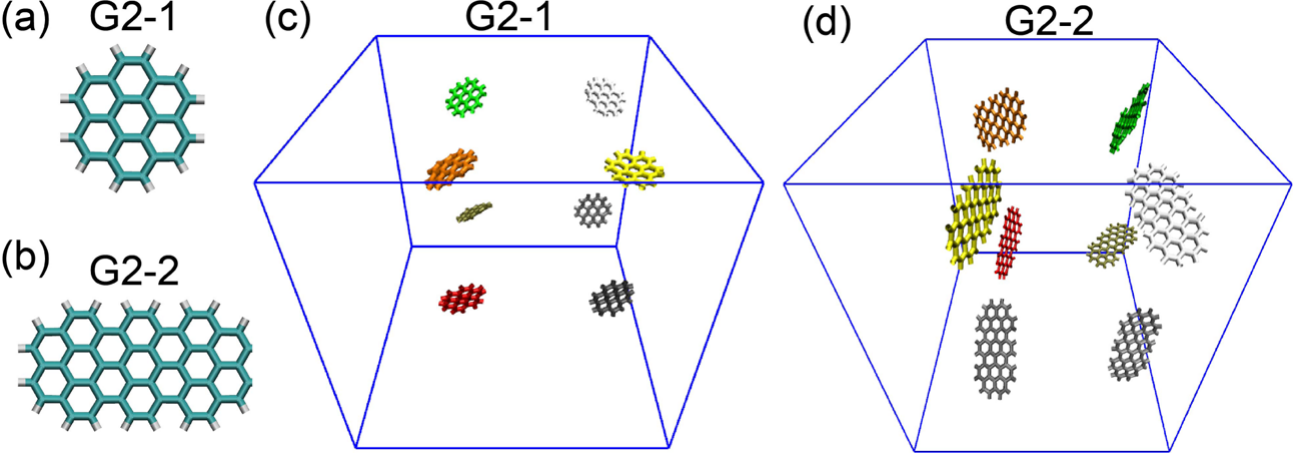
Structures of the two molecules studied in this research: (a) **G2**–**1** graphene flake and (b) **G2**–**2** graphene flake. (c) Initial simulation setup with eight **G2**–**1** graphene flakes placed at the corners of the simulation box. (d) Initial simulation setup with eight **G2**–**2** graphene flakes placed at the corners of the simulation box. Water molecules are not shown in (c) and (d).

The structures of the graphene flakes were generated using the Automated Topology Builder (ATB) online toolkit.^51,52^ Eight graphene molecules were placed at the corner grid node inside a cubic simulation box with a side length of 6.0 nm, so that there are two graphene flakes on each edge, and the distance between adjacent graphene flakes is 3.0 nm. The remaining space was filled with SPC water molecules. There are 6000–7000 water molecules for each simulation, exact values are listed in Table 1, this result in the density of graphene flake to be 2.0–4.0 wt %, similar to the typical graphene density in experiment works, 1.0–4.0 wt %.^1,3^ The simulation setup is illustrated in panels c and d of Figure 1, where the water molecules are not shown for clarity. The cubic simulation box length of 6 nm was selected to be sufficiently large to prevent large graphene aggregates from interacting with their own periodic images. This is particularly important under SEF conditions, where aggregates become extensively elongated in one direction.

**Table 1:** Simulation Parameters for Each Run in This Research. *E*_0_ = 0.0 V/nm Indicates That There is No EF in the System, *f* = 0.0 GHz Indicates That the EF is DC

ID	molecule	EF condition	*E*_0_	*f*	solvent	num. of
			V/nm	GHz		water
1	G2–1	0EF	0.0	-	water	6925
2		SEF	2.0	0.0	water	6925
3		AEF	2.0	2.45	water	6925
4		CPEF	2.0	2.45	water	6925
5	G2–2	0EF	0.0	-	water	6252
6		SEF	2.0	0.0	water	6813
7		AEF	2.0	2.45	water	6813
8		CPEF	2.0	2.45	water	6813

It should be noted that for the **G2**–**2** system under the SEF condition, the simulation box dimensions were modified to 12 nm × 4 nm × 4 nm. This adjustment was made to prevent the aggregate from interacting with its periodic image along the *x*-direction. As will be discussed in Sections 3.2.3 and 3.2.4, under SEF, the **G2**–**2** aggregate adopts a stretched configuration elongated along the *x*-direction. The larger box dimension in the *x*-direction ensures that such interactions are avoided. Representative snapshots of aggregates within their simulation boxes are provided in Figure S9. These confirm that the chosen box sizes are sufficiently large to prevent finite-size artifacts arising from the interaction of an aggregate with its own periodic image.

In the MD simulation, van der Waals (VdW) interactions between atoms are governed by Lennard-Jones potential

1 $$V_{LJ}=4\epsilon_{ij}\left[{\left(\frac{\sigma_{ij}}{r_{ij}}\right)}^{12}-{\left(\frac{\sigma_{ij}}{r_{ij}}\right)}^{6}\right]=\frac{C_{12,ij}}{r_{ij}^{12}}-\frac{C_{6,ij}}{r_{ij}^{6}}$$

and the electrostatic interactions are calculated using the Coulomb potential

2 $$V_{Col}=\frac{q_{i}q_{j}}{4\pi \epsilon_{0}r_{ij}}$$

In the equations above, *r*_*ij*_ indicates the distance between atoms *i* and *j*, and *q*_*i*_ is the partial charge of atom *i*. Bonded interactions between carbon atoms in graphene were modeled using harmonic potentials. All bonded and nonbonded parameters (*C*_12,*ij*_ and *C*_6,*ij*_) were obtained from the GROMOS 54A7 force field,^53^ which is parametrized and suitable for simulating hydrocarbon systems. Parameters for these nonbonded and bonded interactions are provided in the Supporting Information. Long-range electrostatic interactions were treated using the particle-mesh Ewald (PME) method, and long-range van der Waals interactions were treated with a cutoff scheme. The Lennard-Jones potential was shifted by a constant to zero at the cutoff distance, ensuring a continuous potential without affecting the force.

For water molecules, we employed the SPC water model.^54,55^ Covalent bonds involving hydrogen atoms in water molecules (O–H bonds) and in graphene (C–H bonds) were constrained using the SETTLE and LINCS algorithms, respectively, to maintain fixed bond lengths during the simulations.^56,57^

Each simulation underwent energy minimization using the steepest descent algorithm, followed by NVT and NPT equilibration phases of 1.0 ns each. The production run was performed using the Velocity-Verlet algorithm with a 2 fs time step under the *NPT* ensemble for 200 ns to ensure system equilibrium and allow for complete observation of the aggregation behavior. It can be shown that 2 fs time step could yield accurate system dynamics and produces results consistent with those obtained using a finer 1 fs (See Figure S12 in Supporting Information). All simulations were conducted using GROMACS 2024 ^58–60^ on a system equipped with 4 Intel i9-12900KF CPU cores and one NVIDIA GeForce RTX 3080 Ti GPU for acceleration. The temperature was maintained at 300 K using the velocity-rescaling thermostat (τ = 0.1 ps), and the pressure was maintained at 1 bar using the Parrinello–Rahman barostat^61,62^ with a time constant of 2.0 ps. System coordinates were saved every 20 ps, resulting in a total of 10,000 frames per simulation for subsequent analysis.

In this work, we explore how different external EFs impact the aggregation of graphene flakes. We investigate four field types: (1) 0EF, where no external EF is applied (amplitude = 0.0 V/nm), serving as a baseline for comparison; (2) SEF, where the field’s strength and direction remain constant throughout the simulation; (3) AEF, where the field direction oscillates along the ± *x* direction over time. The AEF is described by the equation *E*_*x*_(*t*) = *E*_0_ cos­(2π*ft*), where *E*_0_ = 2.0 V/nm is the field amplitude and *f* = 2.45 GHz is the frequency. This amplitude is relatively strong and has been explored in previous simulation and experimental studies.^63–68^ The chosen frequency corresponds to the resonance frequency of water, which is well-studied and commonly applied in microwave ovens.^69^ Other more moderate EF strength and frequencies may result in different behaviors of graphene flake aggregation, and could be a possible future research direction.

The final type of electric field we explored is the CPEF, where the field vector rotates counterclockwise in the *y*–*z* plane when viewed along the–*x* direction, and the equation could be expressed as

3 $$\begin{array}{ll} & E_{x}\left(t\right)=0\\ & E_{y}\left(t\right)=E_{0}\;\cos\left(2\pi ft\right)\\ & E_{z}\left(t\right)=E_{0}\;\cos\left(2\pi ft-\pi /2\right) \end{array}$$

Note that the GROMACS source code required modification to implement an electric field with the specific phase relationship defined for the CPEF, as shown in eq 3. Simulations for the different graphene flakes, along with their associated solvent and EF conditions, are listed in Table 1. Topology files for the molecules and the force field parameters (gromos54a7_atb.ff) are provided in the Supporting Information (Supporting Information).

It is worth noting that the force field used in this work operates under an electrostatic assumption, where the partial atomic charges are fixed and remain unaffected by the external EF. Consequently, the EF does not induce extra intramolecular dipole moment on individual graphene or water molecules. However, the field does exert a torque on the permanent dipole moments of water molecules, aligning them collectively. This alignment induces a macroscopic solvent polarization, creating an indirect, field-mediated solvent effect. This approach allows us to isolate and specifically investigate the role of this polar solvent response in modulating the aggregation process, independent of intramolecular charge redistribution.

### 2.2 Aggregation Metrics

As graphene flakes have a discotic, flat shape, their mutual interactions depend on their relative orientations and configurations. For instance, when two flakes are stacked parallel to each other, their binding is strong due to π–π interactions; other configurations, such as T-shaped, offset-stacked, or edge-to-edge interactions, are significantly weaker.^10,70^ To differentiate between these binding configurations, we utilize several metrics to measure the intermolecular distances between two graphene flakes, large distances indicate that the two molecules are far from each other, thus the interaction is weak, while small distance indicate strong interactions.

Metric A: pairwise minimum distance. The distance between graphene *a* and graphene *b* is defined as

4 $$R_{a,b}^{A}=\min_{i\in a}\min_{j\in b}r_{ij}$$

where *r*_*ij*_ is the Cartesian distance between atom *i* in graphene *a* and atom *j* in graphene *b*. Graphene *a* and *b* are considered bound when *R*_*a*,*b*_^*A*^⩽0.55 nm. For example, in Figure 2, the distance between graphene *a* and *b* under Metric A corresponds to the distance between atoms *a*_3_ and *b*_4_, as this represents the shortest pairwise atomic distance.

**Figure 2:**
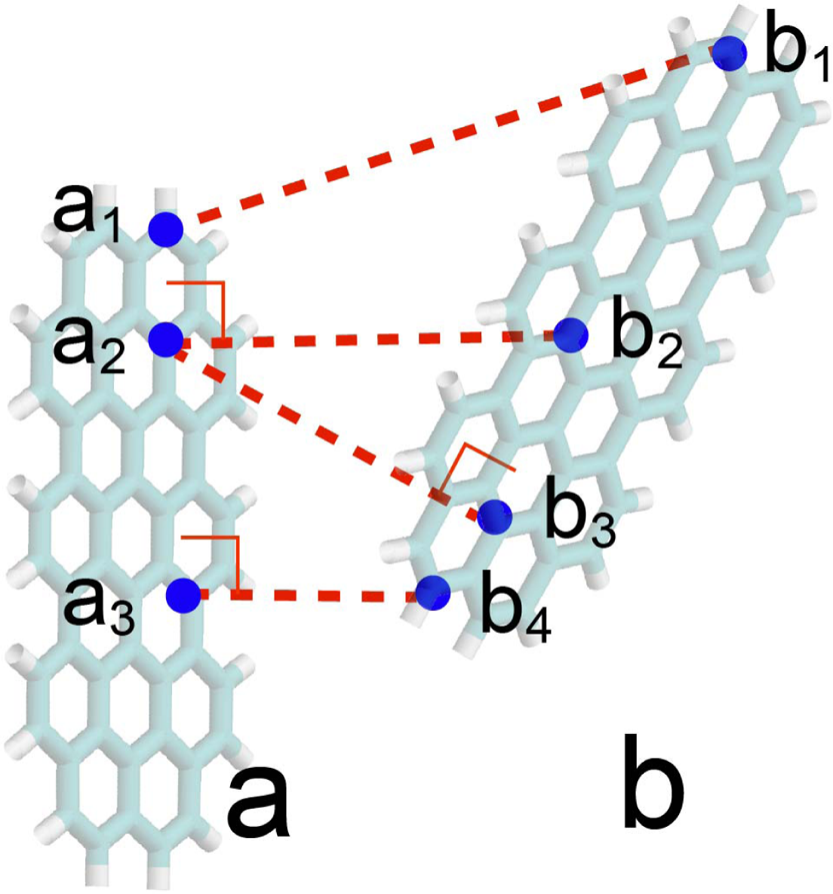
Illustration of the distance metrics between two graphene flakes, *a* and *b*. The Metric A distance *R*_*a*,*b*_^*A*^ equals to the Cartesian distance $\overset{®}{a_{3}b_{4}\;}$ , while the Metric B distance *R*_*a*,*b*_^*B*^ equals to the Cartesian distance $\overset{®}{a_{1}b_{1}\;}$ . Furthermore, the minimum distance from graphene *a* to atom *b*_2_ is $\overset{®}{a_{2}b_{2}\;}$ , whereas the minimum distance from graphene *b* to atom *a*_2_ is $\overset{®}{a_{2}b_{3}\;}$ .

**Figure 3:**
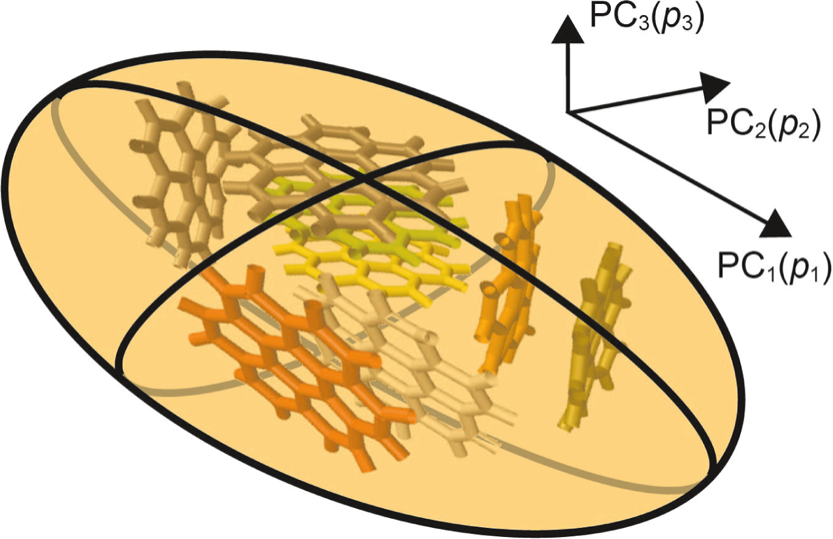
Schematic of Principal Components and Moments. This diagram illustrates the three principal components (PC_*i*_) and their associated moments (*p*_*i*_) for a graphene aggregate, which can be approximated as an ellipsoid. The first principal component (PC_1_, corresponding to the largest moment *p*_1_) indicates the direction of greatest elongation, representing the largest variance. The second principal component (PC_2_, moment *p*_2_) describes the “fatness” of the aggregate, indicating its largest extension along a direction perpendicular to PC_1_. The third principal component (PC_3_, moment *p*_3_) captures the “thickness” of the aggregate, reflecting its elongation along the direction orthogonal to both PC_1_ and PC_2_.

Metric A provides the most lenient criterion for determining binding between two graphene flakes. Most interaction configurations satisfy this criterion, including parallel stacking, offset stacking, T-shaped interactions, and edge-to-edge interactions. Essentially, if any part of one graphene flake is sufficiently close to another, they are identified as bound. Consequently, Metric A alone cannot distinguish strongly bound configurations from weakly bound ones, necessitating an additional metric for this purpose.

Metric B: pairwise maxmin distance.

5 $$R_{a,b}^{B}=\max\left[\left(\max_{i\in a}\min_{j\in b}r_{ij}\right),\left(\max_{i\in b}\min_{j\in a}r_{ij}\right)\right]$$

In the first term within the square brackets, *r*_*ij*_ denotes the distance between atom *i* in graphene *a* and atom *j* in graphene *b*. The max min operator first identifies the shortest distance from atom *i* in graphene *a* to any atom in graphene *b*, and then selects the maximum value among these minimum distances. The second term in the brackets is analogous to the first, with the roles of *a* and *b* swapped. A final max operator outside the brackets selects the larger value from the two max min results.

The two terms within the square brackets are both necessary because they are not commutative upon interchanging *a* and *b*. For example, in Figure 2, the minimum distance from graphene *a* to atom *b*_2_ is $\overset{®}{a_{2}b_{2}\;}$ , whereas the minimum distance from graphene *b* to atom *a*_2_ is $\overset{®}{a_{2}b_{3}\;}$ . The value of *R*_*a*,*b*_^*B*^ is $\overset{®}{a_{1}b_{1}\;}$ , as this represents the minimum distance from graphene *a* to atom *b*_1_ and is also the maximum among all such minimum distances.

Figure 2 illustrates that graphene flakes *a* and *b* interact weakly; only atoms *a*_3_ and *b*_4_ are in close proximity, while other regions (e.g., atoms *a*_1_ and *b*_1_) remain distant and do not contribute significantly to the interaction. Under this configuration, the calculated Metric A distance *R*_*a*,*b*_^*A*^ is small, indicating that *a* and *b* are interacting to some extent, but it does not quantify the binding strength. In contrast, the Metric B distance *R*_*a*,*b*_^*B*^ is relatively large, reflecting the weak overall interaction. A small *R*_*a*,*b*_^*B*^ value occurs only when the two flakes are parallel-stacked. We therefore use the criterion *R*_*a*,*b*_^*B*^⩽ 0.75 nm to determine whether two graphene flakes are strongly bound (i.e., parallel-stacked). Similar metrics to calculate intermolecular distances were used in our previous works.^36,37,71^

It should be noted that the thresholds for defining Metric A and B bonds (0.55 and 0.75 nm, respectively) were determined via a sensitivity analysis. As shown in Figure S6, the identification of these bonds is robust to small deviations in the selected cutoff distances.

During the simulation, graphene flakes bind to each other and form large aggregates. A graphene flake is considered part of an aggregate if it is bound to at least one other molecule within that aggregate. To characterize the size of these aggregates, we use the mass-averaged aggregation number *g*_2_ as a function of time, as it is directly related to mass spectrometric measurement in experiments,^72–74^ which is defined as

6 $$g_{2}\left(t\right)=\frac{\sum_{i}n_{i}\left(t\right)i^{2}}{\sum_{i}n_{i}\left(t\right)i}$$

where *t* is the simulation time, *n*_*i*_(*t*) is the number of aggregates containing *i* molecules in the simulation box at time *t*, and binding between graphene flakes can be determined using either Metric A or Metric B, resulting in distinct *g*_2_ values denoted as *g*_2_^*A*^ and *g*_2_^*B*^, respectively.

We chose to analyze the mass-averaged aggregate size, *g*_2_, rather than the number-averaged size, i̅, because *g*_2_ provides a more direct comparison to experimental techniques like SANS and SAXS, particularly for systems containing large aggregates.

We also calculate the size of the largest aggregate as

7 $$g_{\infty }\left(t\right)=\lim_{k\to \infty }\frac{\sum_{i}n_{i}\left(t\right)i^{k}}{\sum_{i}n_{i}\left(t\right)i^{k-1}}=\max\left(i\right)$$

*g*_*∞*_^*A*^ denotes the size of the largest aggregate according to the Metric A criterion, while Metric B characterizes the size of the parallel-stacked aggregate, which typically forms a 1D rod-like structure, so *g*_*∞*_^*B*^ denotes the size of the largest 1D rod-like aggregate. Because the Metric A criterion is looser than that of Metric B, *g*_*∞*_^*A*^ is always greater than or equal to *g*_*∞*_^*B*^.

### 2.3 Low Dimensional Description of the Aggregate Configuration

The resulting self-assembled configuration depends on both the shape of the flakes and the applied external EF conditions. To characterize the aggregate shape, we use the three principal moments of the gyration tensor as low-dimensional collective variables.

For an aggregate with *N* identical carbon atoms, its gyration tensor **S** is defined as follows

8 $$S_{ij}=\frac{1}{N}\sum_{k=1}^{N}r_{i}^{\left(k\right)}r_{j}^{\left(k\right)}$$

where *r*_*i*_^(*k*)^ denotes the *i*-th Cartesian coordinate of the position vector **r** ^(*k*)^ for the *k*-th particle, with the center of mass of the system defined at the origin of the coordinate system, satisfying the condition $\sum_{i=1}^{N}r^{\left(i\right)}=0$ .^75–80^ The gyration tensor **S** has three eigenvectors and associated eigenvalues (principal moments) *p*_*i*_, where *i* = 1, 2, 3, and each *p*_*i*_ represents the variance of the data along the direction of the *i*-th principal component.

As shown in Figure 3, *p*_1_ is typically greater than *p*_2_ and *p*_3_, characterizing the elongation of the graphene aggregate along the direction of the first principal component. In contrast, *p*_2_ describes the “fatness” of the aggregate configuration, representing its extension along the second principal direction (PC_2_), which is perpendicular to the first (PC_1_). Meanwhile, *p*_3_ captures the “thickness” of the aggregate, reflecting its elongation along the third principal component, orthogonal to the first two PCs. For the calculation of *p*_1_, *p*_2_, and *p*_3_, only the coordinates of the heavy carbon atoms are considered, as the terminal H atoms are significantly lighter, and will be ignored, and the aggregate is identified using either Metric A or B.

### 2.4 Free Energy Landscape

Upon obtaining the low-dimensional collective variable $\xi \in R^{M}$ , the Gibbs free energy (FE) landscape of the molecular configuration represented by ξ can be computed as follows

9 $$FE\left(\xi \right)=-k_{B}T\;\log\rho \left(\xi \right)+C$$

In this equation, *k*_B_ represents the Boltzmann constant, *T* is the temperature, and ρ­(**ξ**) is the probability density distribution of the sampled data at the location of ξ. The constant *C* is arbitrary.

In this research, the probability densities ρ­(**ξ**) are determined using a binning approach: the low-dimensional space is partitioned into equal-sized square bins, and the number of sampling points within each bin is counted. The probability density is then calculated as the ratio of the counts to the total number of samples, divided by the area of a single bin. High density values and corresponding low free energy values FE­(**ξ**) indicate greater stability in the molecular configuration, whereas elevated free energy values suggest reduced stability. As the first two principal components (p1 and p2) capture the majority of the variance (with eigenvalues significantly larger than the third), and contain more information, we use them for dimensionality reduction, to make the interpretation clearer. In this work, we explore the aggregation free energy landscape spanned by only top two principal moments, i.e., ξ = (*p*_1_, *p*_2_).

It is important to note that the free energy, FE­(ξ), calculated for the 0EF and SEF conditions represents a true equilibrium Gibbs free energy, as these systems can reach a steady state. In contrast, under the AEF and CPEF conditions, the system remains out of equilibrium due to the continuously changing field direction and strength. Consequently, the FE­(ξ) for these cases should be interpreted as an effective quasi-free energy landscape, which characterizes the relative stability of aggregate configurations under the applied nonequilibrium driving force.

## 3 Results and Discussions

### 3.1 G2–1

#### 3.1.1 The Bond Number in Aggregates

We use two different metrics to characterize the binding properties of graphene flakes: Metric A, which is looser and captures all types of molecular interactions, and Metric B, which is stricter and captures only strong bindings between flakes. Figure 4a–d displays the time evolution of the number of Metric A and Metric B bonds under four EF conditions. Initially, in each simulation, no bonds are present, and both Metric A and Metric B bond counts are zero. As time progresses, the number of bonds increases and eventually reaches a plateau, fluctuating around an average value after the simulation equilibrates at approximately 20 ns.

**Figure 4:**
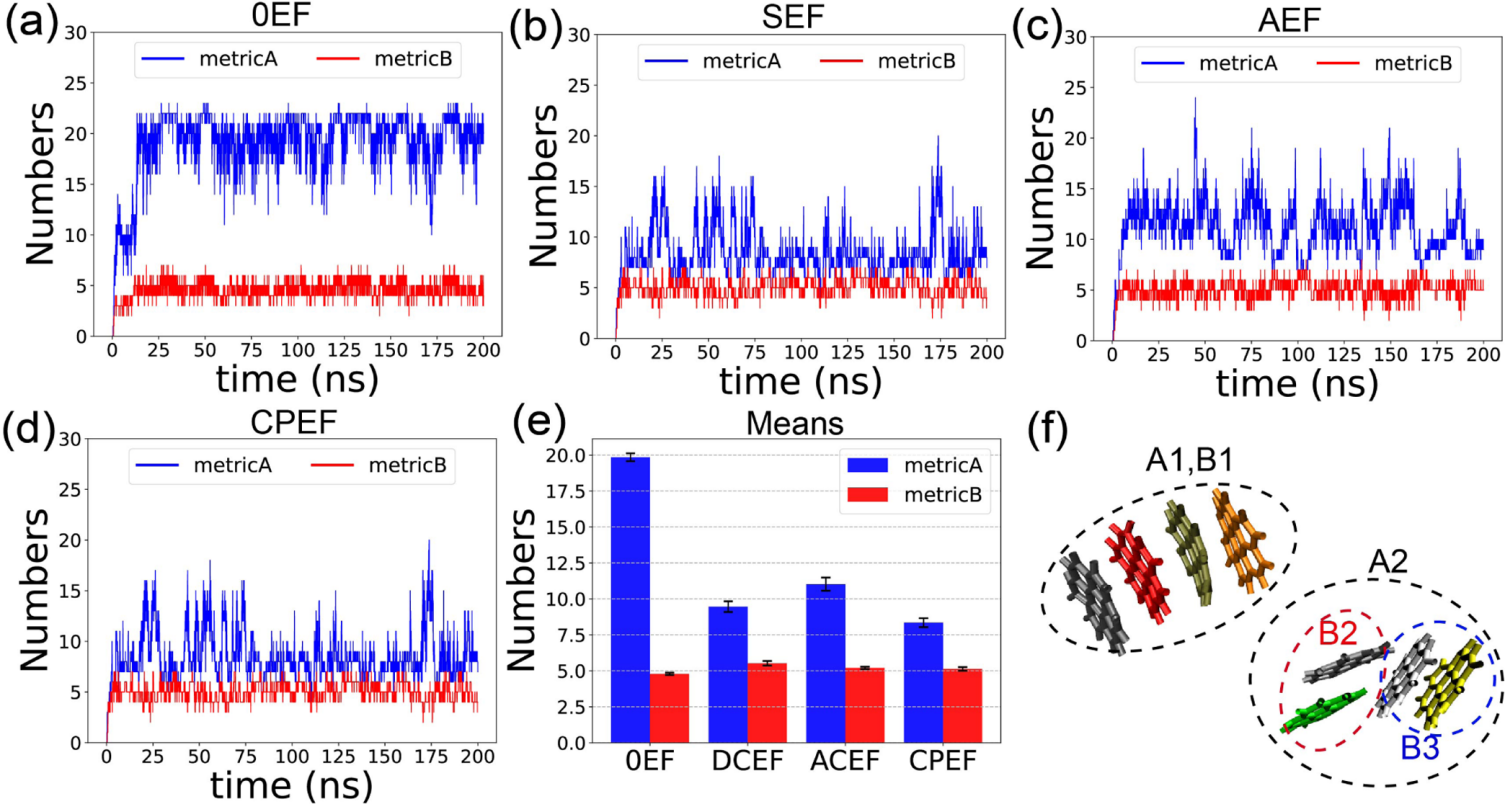
Time evolution of the number of bonds in the **G2**–**1** aggregate under different EF conditions: (a) 0EF, (b) SEF, (c) AEF, and (d) CPEF. (e) Average bond numbers calculated from the equilibrated portion of the simulation trajectory. (f) Representative snapshot illustrating the identification of aggregates based on Metric A and Metric B criteria.

The mean values of Metric A and Metric B bond numbers were calculated using data from the equilibrated period. These values are displayed as a bar plot in panel e of Figure 4. We can observe that under all EF conditions, the number of Metric A bonds is consistently greater than that of Metric B bonds. This is expected, as Metric A employs a looser criterion for determining binding between graphene flakes compared to Metric B.

In Figure 4e, we can also observe that the number of Metric B bonds remains relatively similar across different EF conditions, whereas the number of Metric A bonds varies more significantly. In the absence of an external EF, there are approximately 20 Metric A bonds. When an external EF is applied, the number of Metric A bonds decreases, indicating that the EF suppresses weak interactions between graphene flakes. In contrast, the impact on strong parallel stacking interactions (captured by Metric B) is less pronounced.

Figure 4f provides an example illustrating how Metric A and Metric B differ in judging intermolecular interactions, leading to different aggregate identifications. The eight graphene flakes are colored differently and form two distinct groups: one comprising four graphenes on the left and another four on the right. Since these two groups are spatially separated and do not interact, only two aggregates-A1 and A2, marked by black dashed circles-are identified under the Metric A criterion.

When applying the Metric B criteria, molecules within the left group (A1) are all strongly bound through π–π stacking and are therefore identified as a single Metric B aggregate (B1). In the right group, however, there are two parallel-stacked subgroups (B2 and B3). Molecules within each subgroup are strongly stacked, but molecules from different subgroups are not considered bound under the Metric B criterion. Consequently, the eight graphene flakes are partitioned into three groups under Metric B: B1, B2, and B3, marked by black, red, and blue dashed circles, respectively.

#### 3.1.2 EFs Impact Aggregate Sizes

Using the Metric A and Metric B criteria, we can identify the number of aggregates and calculate both the average aggregate size using eq 6 and the size of the largest aggregate via eq 7. The time evolution of these values is shown in Figure 5, where panels a-d correspond to different EF conditions.

**Figure 5:**
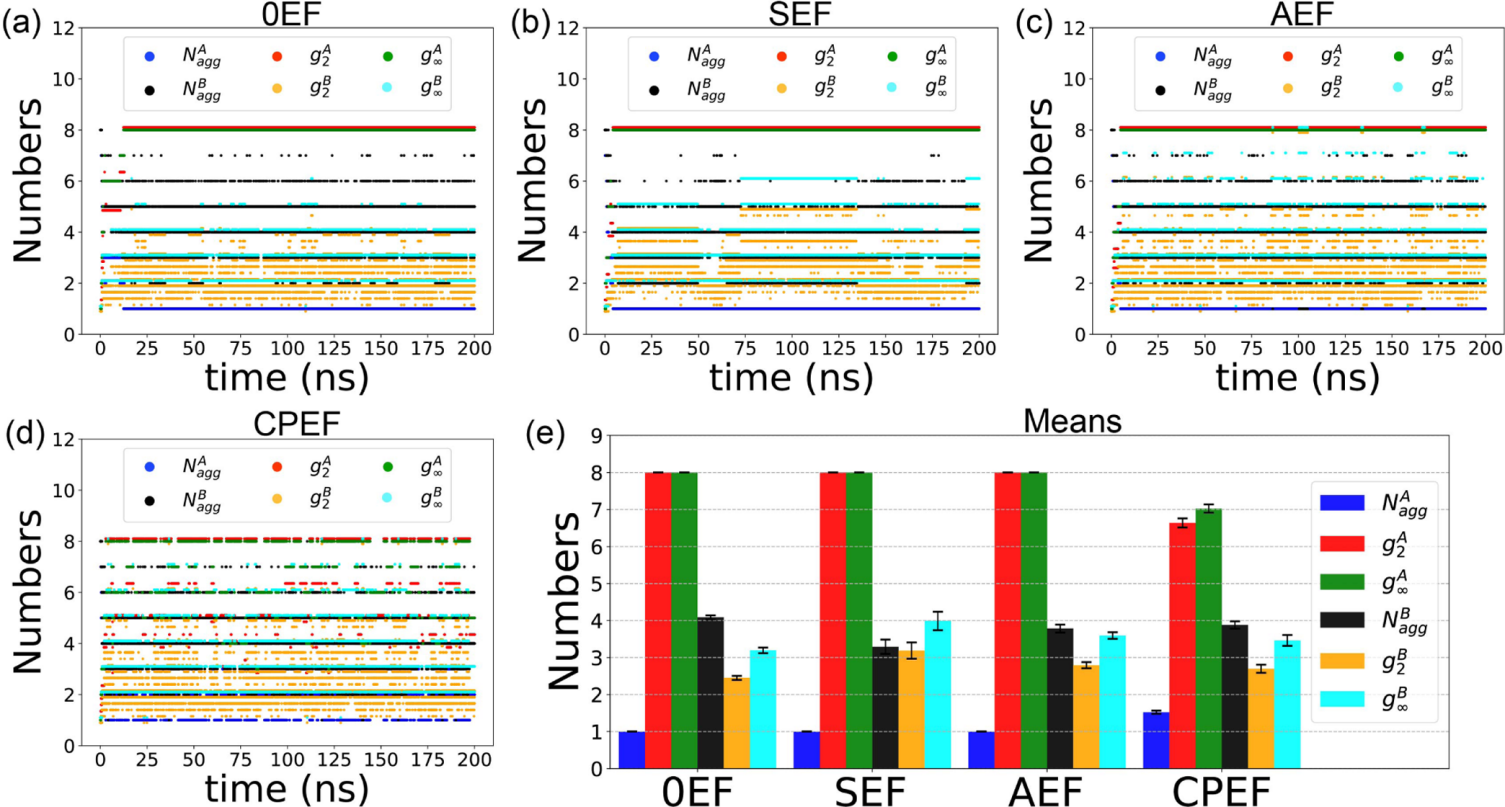
Time evolution of the number of **G2**–**1** aggregates and their sizes (based on Metric A/B) for the following conditions: (a) 0EF, (b) SEF, (c) AEF, and (d) CPEF. (e) Average number of aggregates and average aggregate sizes under the different EF conditions.

We observe that the number of aggregates under both metrics, *N*_agg_^*A*^ and *N*_agg_^*B*^, starts at 8 at the beginning of the simulation and decreases over time. This initial condition occurs because each graphene flake is initially far from the others and is not bound under either metric; thus, each molecule is identified as a separate aggregate. Consequently, the average aggregate size (*g*_2_^*A*^, *g*_2_^*B*^) and the size of the largest aggregate (*g*_*∞*_^*A*^, *g*_*∞*_^*B*^) all begin at 1. These values subsequently increase and eventually reach a plateau once the system equilibrates.

To facilitate a clearer comparison of these values, the mean values were calculated using data from the equilibrated portion of the simulation (*t* > 20 ns). These means are displayed as a bar plot in panel e of Figure 5, with error bars representing the standard error of the mean (SEM). The four groups of bars correspond to the four EF conditions, and each calculated value is distinguished by a different color.

From Figure 5e we can see that *N*_*agg*_^*A*^ is 1 under 0EF, SEF and AEF conditions, and their *g*_2_^*A*^,*g*_*∞*_^*A*^ values are all 8, this means that at all 8 graphene flakes are self-assembling into one single aggregate, even though not every pair of graphene flakes are binded (other wise there will be 8 × 7/2 bonds, which is larger than what we see in Figure 4), since metric A has a loose criteria, all graphene molecules are directly or indirectly connected and belongs to a single aggregate, the size of this aggregate is 8. The exception is shown for the CPEF condition, under this EF, the number of metric A aggregate is greater than 1, the average size and the largest size are both smaller than 8, this means that graphene flakes could not form a single aggregate, CPEF could weaken the interactions between graphenes, thus increase the aggregate number and decrease the aggregate sizes.

When examining the Metric B aggregate number and sizes in panel e, we observe that the aggregate count *N*_agg_^*B*^ is larger than *N*_agg_^*A*^, while the aggregate sizes are correspondingly smaller. This result arises because Metric B employs stricter criteria than Metric A; only graphene flakes exhibiting strong interactions are identified as bound, leading to the identification of a greater number of smaller aggregates. Comparing different EF conditions reveals that under 0EF, the number of Metric B aggregates (*N*_agg_^*B*^) is the largest, and the aggregate sizes are smaller than under conditions where an EF is applied. This indicates that the application of an EF promotes the formation of strong bindings between graphene flakes, resulting in a smaller number of longer, one-dimensional rods formed through parallel π–π stacking.

It is worth noting that the time evolutions of (*N*_agg_, *g*_2_, *g*_*∞*_) values in Figure 5 exhibit rapid oscillations due to the fast, dynamic binding and dissociation of graphene flakes. These fluctuations cause the measured quantities to change quickly over very short time scales. Because each plotted data point has a finite size, and these transitions occur more rapidly than the visual resolution of the figure, the points appear to overlap along the time axis, which can mask the instantaneous jumps in value.

#### 3.1.3 The Alignment of Aggregates by EF

Graphene flakes self-assemble into a single aggregate under most EF conditions. To characterize how the EF aligns this aggregate, we first obtain the principal components, particularly the first principal component (PC_1_), which indicates the direction of aggregate elongation. Since the EF is applied along the *x*-axis for the 0EF, AEF, and SEF conditions, we calculate the angle θ between the *x*-axis and PC_1_, as illustrated in the inset of Figure 6a. It can be demonstrated that for a uniformly oriented vector on a 2D sphere (the proof is provided in the Supporting Information), the corresponding single variable cos θ follows a uniform distribution. Therefore, we use cos θ, specifically its absolute value | cos θ|, to quantify the alignment of the graphene aggregate by the EF. The absolute value ensures a non-negative result and is equivalent to flipping PC_1_ if it points in the–*x* direction.

**Figure 6:**
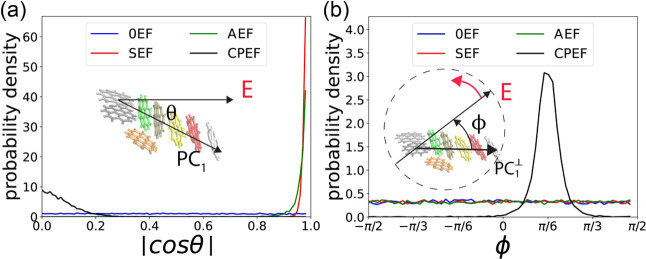
Alignment of the **G2**–**1** aggregate by the electric field. (a) Probability density distribution of | cos θ|, where θ is the angle between the first principal component (PC_1_) and the direction of the applied EF. (b) Probability density distribution of ϕ, the angle in the *y*–*z* plane between PC_1_^⊥^ (the projection of PC_1_ into the *y*–*z* plane) and the rotating CPEF vector.

The probability density distribution of | cos θ| for the four EF conditions is shown in Figure 6a. When no EF is applied (0EF), as indicated by the blue curve, | cos θ| exhibits a uniform distribution, signifying that the aggregate is randomly oriented in space and its PC_1_ is uniformly distributed on the 2D sphere. In contrast, when an SEF is applied, a sharp peak appears at | cos θ| = 1.0 (i.e., θ = 0), demonstrating that the SEF aligns the aggregate such that it elongates along the direction of the applied field.

This phenomenon appear because of the polarity property of water molecules, under SEF, water molecules will be oriented so that their dipole moment against (antiparallel with) the EF, this will create directional hydrogen bond network, and 1D water nano wire in the direction of EF.^43–50^ Elongated graphene aggregate will be aligned parallel to the EF, as this can minimize the breaking of hydrogen bond network.^43,44^ The demonstration of the 1D hydrogen bond structure could be seen in Figure S7 in the Supporting Information.

When an AEF is applied, a peak remains at | cos θ| = 1.0, but its height is significantly lower than in the SEF case. This occurs because the AEF is also directed along the ± *x* axis and can align the aggregate in the *x* direction. However, since the field direction oscillates continuously, its effective strength is reduced to $\frac{\sqrt{2}}{2}E_{0}$ even though the amplitude *E*_0_ = 2.0 V/nm is the same as that of the SEF. This reduction in effective strength diminishes its ability to align the graphene aggregate compared to the static field.

When the CPEF is applied, a lower peak appears at | cos θ| = 0.0 (θ = π/2), indicating that the elongation of the graphene aggregate is perpendicular to the *x*-axis. This alignment occurs because the CPEF is applied in the *y*–*z* plane, as described in Section 2.1, causing the aggregate to elongate within that plane and thus perpendicularly to the *x*-axis.

To track how the aggregate alignment is influenced by the CPEF and compare it with other EF conditions, we calculate the angle ϕ between the CPEF vector E⃗ and PC_1_^⊥^, which is the projection of PC_1_ onto the *y*–*z* plane. As illustrated in the inset of Figure 6b, E⃗ rotates counterclockwise in the *y*–*z* plane when viewed against the *x*-axis. The angle ϕ is measured starting from PC_1_ toward E⃗, such that a positive ϕ indicates that PC_1_^⊥^ lags behind the CPEF in the rotational direction, while a negative ϕ would indicate that PC_1_^⊥^ leads E⃗. For the 0EF, SEF, and AEF conditions, where no E⃗ component exists in the *y*–*z* plane, we instead calculate the angle between PC_1_^⊥^ and the *y*-axis.

It should be noted that while the peak in | cos θ| is centered at 0, indicating the graphene aggregate (represented by PC_1_) is predominantly perpendicular to the *x*-axis, the peak exhibits a finite width. This signifies that PC_1_ is not perfectly static; it undergoes fluctuations, occasionally deviating from the *y*–*z* plane. These deviations directly influence the measured angle ϕ between PC_1_ and the electric field E⃗. To isolate the relevant orientational response from this fluctuation and consistently track the alignment relative to the field, we instead calculate ϕ as the angle between the projection of PC_1_ onto the *y*–*z* plane (denoted PC_1_^⊥^) and the field E⃗.

From Figure 6b, we observe that ϕ exhibits a uniform distribution for the 0EF, SEF, and AEF conditions, indicating that the graphene aggregate’s orientation in the *y*–*z* plane is unaffected by the applied field in these cases. In contrast, under CPEF, a significant peak emerges centered at ϕ ≈ + π/6. This result demonstrates that as the E⃗ vector of the CPEF rotates around the *x*-axis within the *y*–*z* plane, the graphene aggregate also rotates but consistently lags behind the field vector by an angle of approximately π/6. When the frequency of the CPEF is reduced to 1.2 GHz, the rotation of the EF and the graphene aggregate will both be slow down, the lag angle is smaller than π/6, the synchronization will also be stronger. (See Figure S8).

It should be noted that for the CPEF case, after the system reaches equilibrium, the graphene flakes do not always form a single aggregate of size 8. As shown in Figure 5e, multiple smaller aggregates may coexist in the simulation box. These configurations are excluded from the calculation of PC_1_ and the angles θ and ϕ in this section; only the aggregate containing all eight graphene flakes is considered for these analyses. When the frequency of the CPEF is reduce, for example, to 1.2 GHz, the graphene aggregate could remain as a single structure, and will not dissociate during the whole simulation period (Figure S8).

#### 3.1.4 EF Impacts Configurations of Aggregates

In this section, we discuss how external EFs influence the configuration and shape of graphene aggregates by analyzing the distribution of the principal moments *p*_1_, *p*_2_, and *p*_3_. Since these three moments exist in a three-dimensional space, we project them onto the two-dimensional space spanned by (*p*_1_, *p*_2_) to simplify visualization and interpretation. The third component, *p*_3_, is represented by the color of each data point. As shown in Figure 7, each point corresponds to an aggregate configuration extracted from the simulation trajectory after equilibrium (*t* > 20 ns).

**Figure 7:**
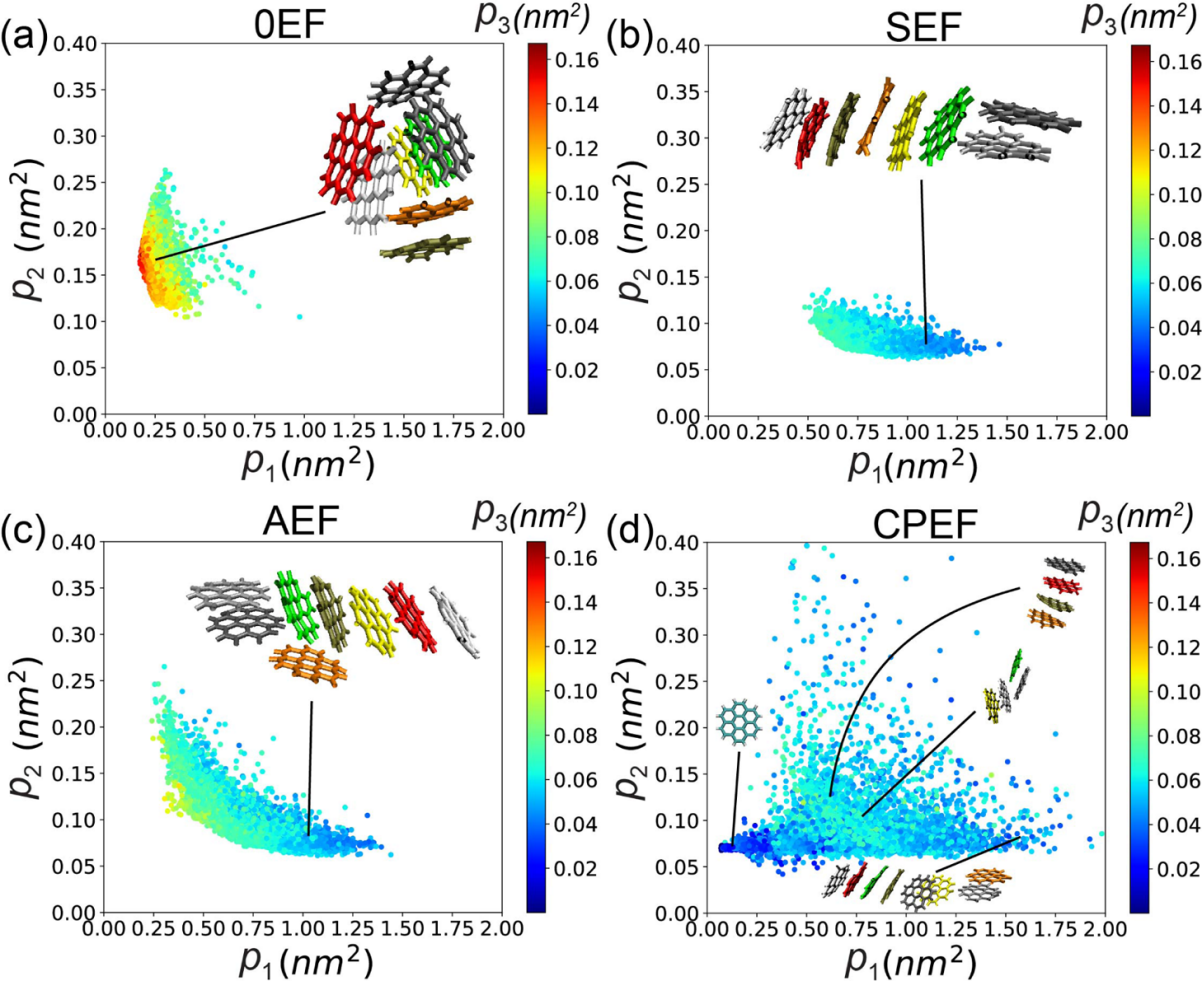
Distribution of **G2**–**1** aggregate configurations in the low-dimensional space defined by the principal moments (*p*_1_, *p*_2_) under the following conditions: (a) 0EF, (b) SEF, (c) AEF, and (d) CPEF. Each data point is colored according to the value of the third principal moment, *p*_3_.

In Figure 7a, we observe that all data points are located on the left-hand side of the plot, where *p*_1_ values are small, *p*_2_ values are medium, and *p*_3_ values are large (indicated by the red color). This indicates that in the absence of an applied EF, graphene flakes tend to form a globular aggregate configuration with a small aspect ratio and a round-like shape. This is visually confirmed by the representative configuration shown in the inset of panel a, which was extracted from the simulation.

When an SEF is applied, the data point cloud shifts downward and is located in the central lower region, characterized by larger *p*_1_ values and smaller *p*_2_ and *p*_3_ values. This indicates that the SEF stretches the graphene aggregate along the direction of PC_1_ while reducing its dimensions along the two orthogonal directions (PC_2_ and PC_3_), thereby increasing the aspect ratio of the configuration. The inset in panel b shows that graphene flakes bind through parallel π–π stacking interactions, forming a one-dimensional rod-like structure. In contrast, the configuration in panel a consists of shorter rods that pack together randomly. This structural difference explains the larger *g*_2_^*B*^ value observed under the SEF condition compared to the 0EF condition (Figure 5e).

The stretching observed under SEF results from the electric field-induced formation of one-dimensional water nanostructures and directional hydrogen-bond networks. This elongated aggregate configuration minimizes disruption to the ordered hydrogen-bond network, especially when aligned parallel to the applied field.^43–50^

When an AEF is applied (Figure 7c), the distribution of data points combines characteristics of both the 0EF and SEF conditions. The points are spread over a larger region, indicating that under AEF, the graphene aggregate can adopt both elongated one-dimensional rod-like structures and globular, round-like configurations.

When CPEF is applied, as shown in Figure 7d, the behavior differs significantly from the previous cases. Data points are distributed across almost the entire region, including the bottom-left area where all *p*_1_, *p*_2_, and *p*_3_ values are small. This occurs because, under CPEF, the aggregate no longer remains as a single large structure; larger aggregates can split into smaller fragments. These smaller fragments are identified as individual aggregates that are not bound to others. Such small aggregates-particularly individual graphene flakes-exhibit small principal moments and are located in the lower-left region of the plot together with their blue coloration.

In addition to the presence of small aggregates under the CPEF condition, the extremely wide distribution of data points indicates that the aggregates can explore a broader range of configurations. For instance, in the far right region of the figure, the configuration is highly extended. In such cases, graphene flakes are not exclusively bound through π–π stacking interactions but may also exhibit weaker edge-to-edge connections.

In Figure 7a–c, each data point represents an aggregate of size 8, as under the 0EF, SEF, and AEF conditions, graphene flakes strongly attract each other through hydrophobic interactions, π–π stacking, and other forces, enabling them to form a single aggregate containing all building blocks. In contrast, under CPEF, interactions between graphene flakes are disrupted by the field, resulting in smaller aggregate fractions. To illustrate how aggregates of different sizes are distributed in the (*p*_1_, *p*_2_) space, we color each point according to its aggregate size, as shown in Figure 8.

**Figure 8:**
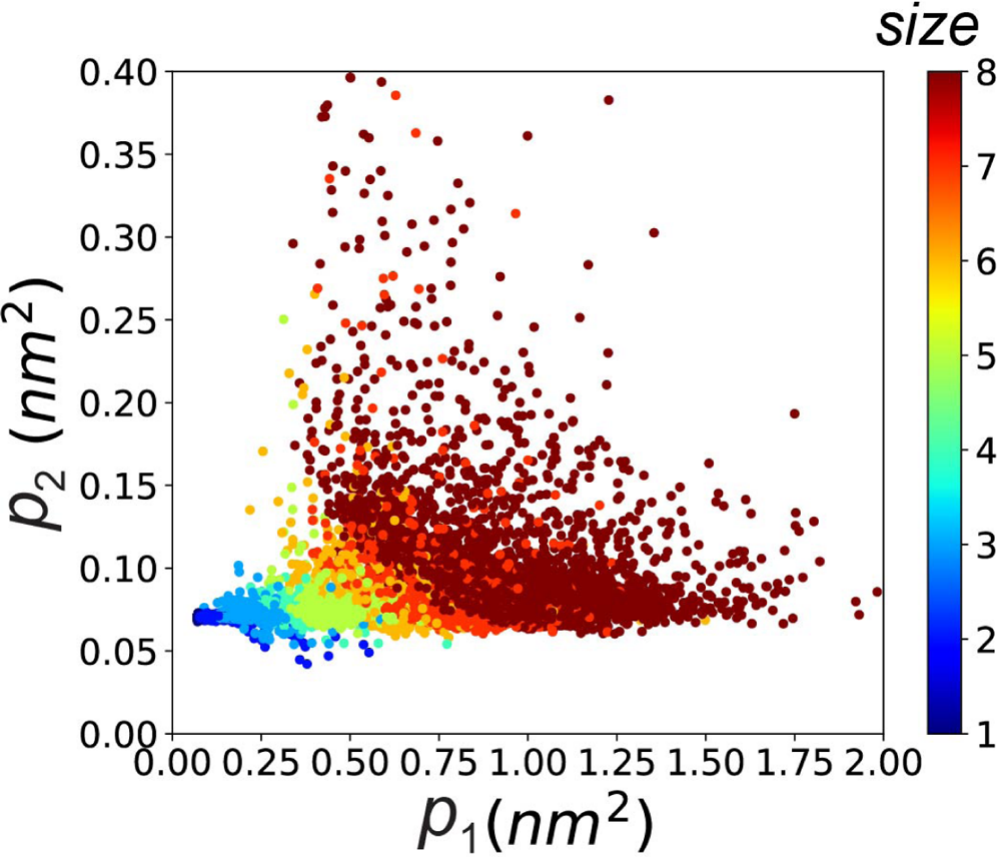
Distribution of **G2**–**1** aggregate configurations in the (*p*_1_, *p*_2_) space under the CPEF condition. Each data point is colored according to the aggregate size.

From Figure 8, we observe that in the lower-left corner, the aggregate size is indeed 1, indicating that even after equilibrium, individual graphene flakes can dissociate from the main aggregate, forming small aggregates of size 1 with correspondingly small principal moments. Progressing from the lower-left to the upper-right region, the aggregate size increases from 1 to 8. Larger aggregates sample a broader region of configurational space due to their greater number of degrees of freedom under CPEF condition.

For other EF conditions, scatter plots of data points in the (*p*_1_, *p*_2_) space, colored by aggregate size, are provided in Figure S2 of the Supporting Information. There, all data points appear red, indicating that the aggregate size is consistently 8 under those conditions.

Figure 7 illustrates how aggregate configurations are distributed in the (*p*_1_, *p*_2_, *p*_3_) space; however, it does not provide information about the stability of these configurations. To understand the stability of different configurations, we use eq 9 to calculate the free energy profile and plot the resulting two-dimensional free energy landscape in the (*p*_1_, *p*_2_) space. Low free energy values indicate stable states where the aggregate remains in a given configuration for extended periods. High free energy regions correspond to unstable configurations that rapidly transition to more stable states.

As shown in Figure 9, panels a-d correspond to the four EF conditions. We observe small dark blue regions in panels a and b, indicating free energy minima that correspond to the most stable aggregate configurations under 0EF and SEF. Under 0EF, the aggregate predominantly maintains a globular state, whereas under SEF, it most frequently adopts a one-dimensional rod-like structure. For the AEF condition, the stable region is broader, signifying that the graphene aggregate configuration can transition more readily between elongated and compact forms. White blank regions in these plots indicate that the free energy are so high, that there is no sampled data.

**Figure 9:**
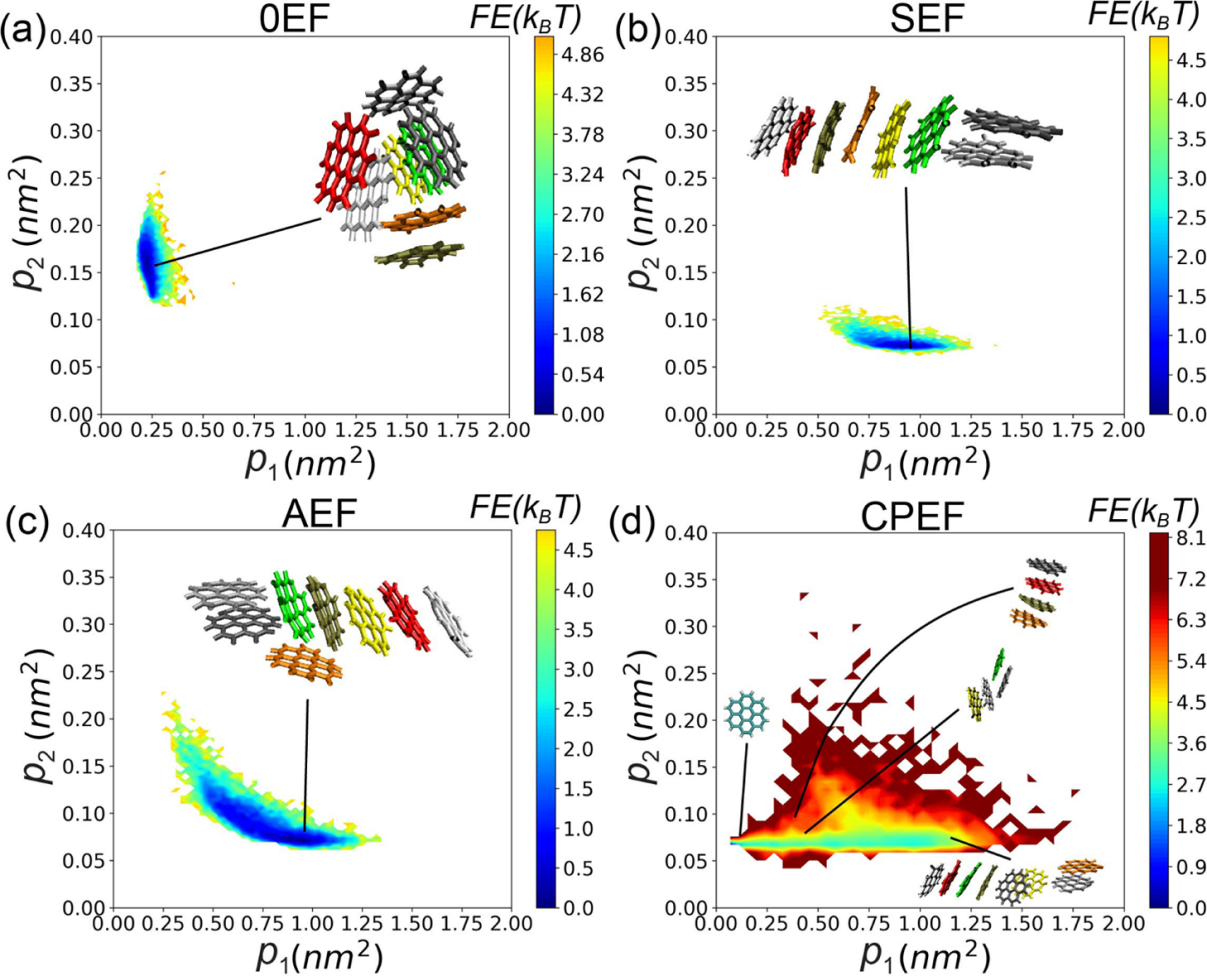
Two-dimensional free energy landscape of the **G2**–**1** aggregate in the principal moment space (*p*_1_, *p*_2_) for the following conditions: (a) 0EF, (b) SEF, (c) AEF, and (d) CPEF.

Under CPEF, although data points sample a wide region in the (*p*_1_, *p*_2_) space as shown in Figure 7d, the actual stable states are not as broadly distributed. As visible in Figure 9d, only a lower strip-shaped region exhibits low free energy, which accommodates the most probable aggregate configurations. Proceeding from left to right along this strip, the aggregate transitions from small, single-flake aggregates to larger, elongated structures. However, all these configurations share small *p*_2_ values, indicating that the aggregates are consistently thin and elongated. In contrast, wider fat configurations with larger *p*_2_ values, which are present under 0EF or AEF conditions, are absent under CPEF. All aggregate fragments, including smaller ones, predominantly adopt a rod-like shape, as illustrated by the snapshot insets in Figure 9d.

It should be noted that all free energy landscapes in Figure 9 are shifted such that the minimum value starts from 0.0 *k*_B_ *T* (at T = 300 K). The color scale is consistent across all panels, meaning that identical free energy values are represented by the same colors, even though the range of the color bar may vary between panels. Moreover, the calculated free energy landscapes are stable and well-equilibrated. As demonstrated in Figure S11 (Supporting Information), the landscape derived from the first half of the simulation trajectory is essentially identical to that obtained from the second half, confirming the robustness and convergence of our analysis.

#### 3.1.5 EF Impact Excluded Volume

The EF also influences the excluded volume of the aggregate. Through hydrophobic interactions, graphene flakes self-assemble to minimize the excluded volume. As shown in Figure 10, each data point is colored according to the excluded volume of the corresponding configuration. In panel a, the excluded volume is relatively small under 0EF, as most points are blue (*V*_ex_ < 4.8 nm^3^) in Figure 10a, under this EF condition, the graphene flakes aggregate primarily through hydrophobic collapse and π–π interactions, forming compact, globular configurations that minimize the system’s excluded volume. However, with the application of SEF or AEF, as shown in panels b and c, graphene aggregate cannot form globular structures, they will be extended in the direction of the EF, this will reduce the hydrophobic collapsing effect, in this case, π–π stacking dominates the interactions among graphene flakes, so the excluded volume will be increased. This is particularly evident for extended rod-like configurations, where the color shifts to yellow and orange (4.8 nm^3^ < *V*_ex_ < 5.2 nm^3^) in Figure 10b,c.

**Figure 10:**
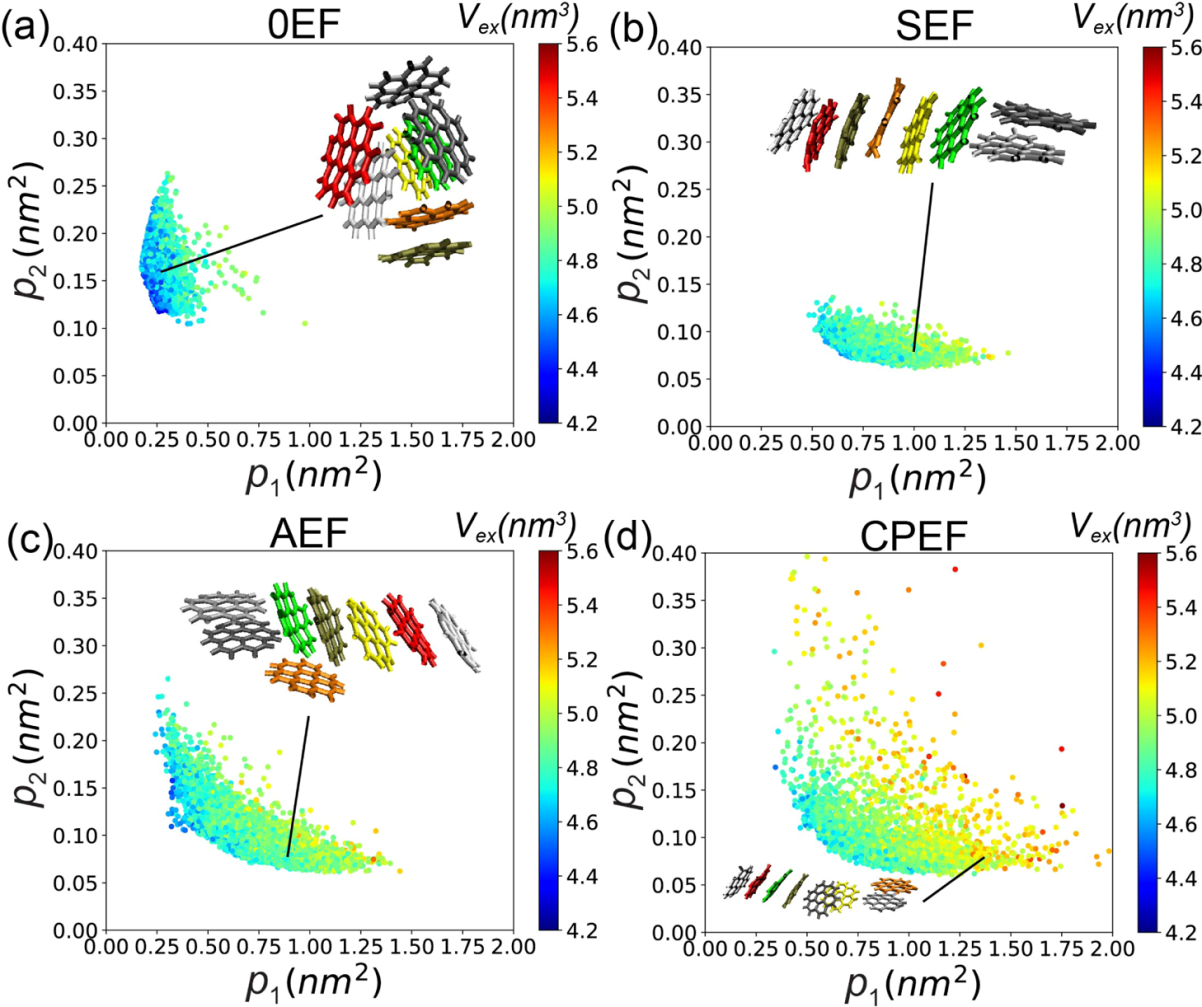
Distribution of **G2**–**1** aggregate configurations in the (*p*_1_, *p*_2_) space, colored by the excluded volume (*V*_*ex*_), for the following conditions: (a) 0EF, (b) SEF, (c) AEF, and (d) CPEF.

Under the CPEF condition, the stability of the graphene aggregate is extensively disrupted, enabling flakes to dissociate more readily. This further diminishes hydrophobic interactions and correspondingly increases the excluded volume, with colors turning to red (*V*_ex_ > 5.2 nm^3^) in Figure 10d. This is particularly evident for larger aggregates of size 8 that adopt extended configurations where the binding between graphene flakes consists of weak edge-to-edge interactions (panel d). These results indicate that the EF, particularly CPEF, counteracts the hydrophobic collapsing effect, thereby increasing the excluded volume of the graphene aggregate.

### 3.2 G2–2

#### 3.2.1 The Bond Number in Aggregates

For the larger graphene flake **G2**–**2**, which also has a greater aspect ratio, the average bond numbers are shown in Figure 11. (The time evolution of bond numbers under different EF conditions is provided in the Supporting Information.) In Figure 11, we observe that the number of Metric A bonds is quite similar across different EF conditions, remaining around 13. Compared to the **G2**–**1** graphene results in Figure 5e, the Metric A bond number under the 0EF condition decreases from 20 to 13 for **G2**–**2**. This reduction indicates fewer pairwise interactions between graphene flakes in the **G2**–**2** aggregate, which results from its more ordered configuration and arrangement of flakes (as visible in the inset of Figure 14a). This ordered structure reduces the number of mutual bindings between graphene pairs.

**Figure 11:**
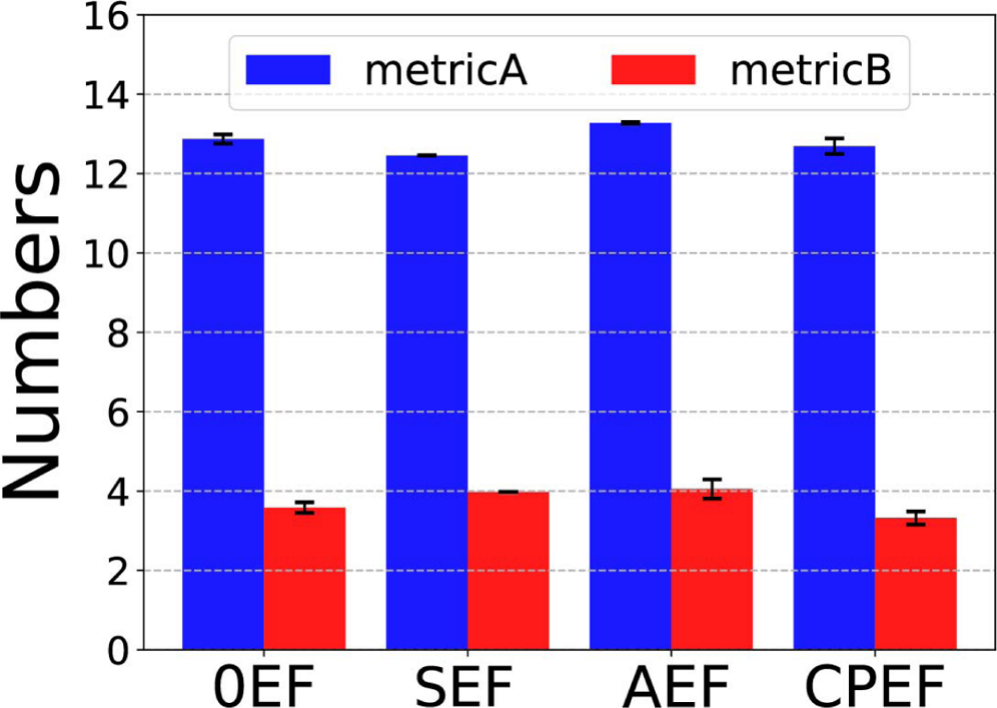
Average number of Metric A and Metric B bonds for the **G2**–**2** graphene aggregate under different EF conditions.

When an EF is applied, the number of Metric A bonds remains significantly higher in the **G2**–**2** case and is similar to the 0EF condition. Although the EF reduces interactions between graphene flakes in the **G2**–**1** aggregate, it has a less pronounced effect on the **G2**–**2** aggregate. This difference arises because **G2**–**2** flakes have a larger size and stronger intrinsic interactions, which can overwhelm the influence of the EF. Consequently, the Metric A bond numbers remain relatively consistent across EF conditions for **G2**–**2**.

Regarding the number of Metric B bonds, as shown in Figure 11, the values are similar across conditions and equal to 4. This is lower than in the **G2**–**1** aggregate, where the Metric B bond number is 5. The reduction occurs because **G2**–**2** has an elliptical shape with a larger aspect ratio, making it more difficult for two flakes to achieve perfect parallel stacking. For instance, if two **G2**–**2** flakes stack with their long axes perpendicular, the Metric B distance *R*_*ab*_^*B*^ remains large, and they are not classified as bound under the Metric B criterion.

#### 3.2.2 EFs Impact Aggregate Sizes

The average number of Metric A/B aggregates and the average aggregate sizes are shown in Figure 12. (The time-dependent values for each frame are provided in Figure S4 of the Supporting Information.) From Figure 12, we observe that the Metric A profiles are identical across all four EF conditions: the aggregate number *N*_agg_^*A*^ is consistently 1, and the aggregate sizes *g*_2_^*A*^ and *g*_*∞*_^*A*^ are both 8, even under CPEF. This indicates that all flakes remain bound under the Metric A criterion. Because **G2**–**2** flakes have larger sizes and stronger pairwise interactions, they bind more readily whether through strong parallel stacking or weak edge-to-edge contacts-resulting in a single aggregate under all field conditions.

**Figure 12:**
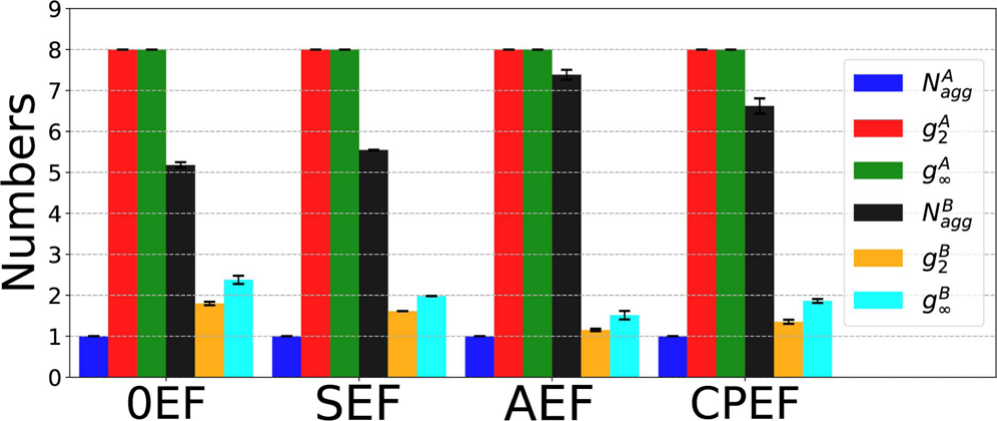
Average number of aggregates and average aggregate sizes for the **G2**–**2** system under different EF conditions.

Regarding the Metric B aggregates, we observe that the 0EF and SEF conditions yield the smallest number of aggregates and slightly larger aggregate sizes. In contrast, the AEF and CPEF conditions increase the number of aggregates while reducing their size. Since only perfectly parallel-stacked graphene pairs are classified as Metric B bonds and contribute to a Metric B aggregate, this behavior indicates that dynamic EFs-such as AEF and CPEF, which vary in magnitude or direction over time-can disrupt these ideally stacked configurations. This disruption results in a greater number of shorter, stacked aggregates.

#### 3.2.3 The Alignment of Aggregates by EF

Similar to the **G2**–**1** case, we obtained the first principal component PC_1_ for **G2**–**2** aggregate and calculated the angle θ between PC_1_ and the *x*-axis. The probability density distribution of | cos θ| derived from the simulation trajectory is shown in Figure 13a. Under 0EF, | cos θ| exhibits a uniform distribution, indicating that the aggregate is randomly oriented in the absence of an EF. When SEF and AEF are applied, sharp peaks appear at | cos θ| = 1.0 (θ = 0.0), with the peak height for SEF being greater than that for AEF. This demonstrates that directional EFs align the aggregate such that its elongation (along PC_1_) coincides with the field direction, and that SEF exerts a stronger aligning effect than AEF due to its higher effective field strength. Under CPEF, a peak occurs at | cos θ| = 0.0 (θ = π/2), indicating that the aggregate aligns perpendicular to the *x*-axis, consistent with the CPEF’s electric field vector E⃗ rotating in the *y*–*z* plane.

**Figure 13:**
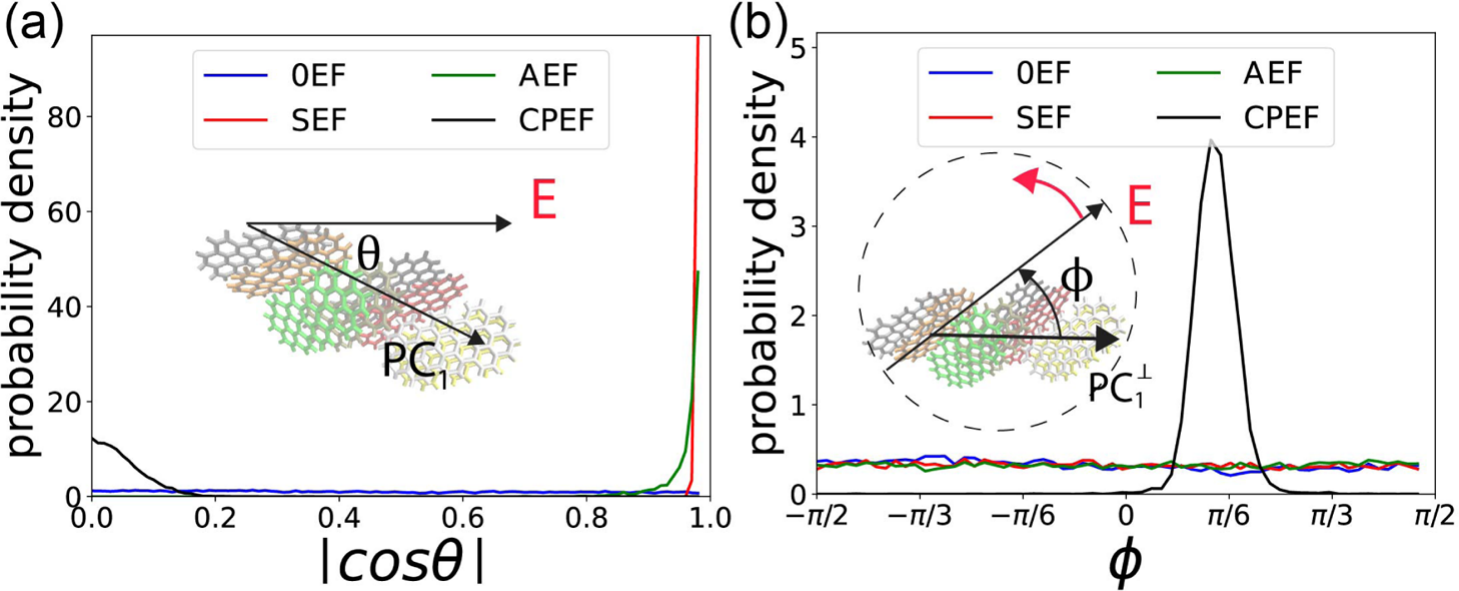
Alignment of the **G2**–**2** aggregate by the EF. (a) Probability density distribution of | cos θ|, where θ is the angle between the first principal component (PC_1_) and the direction of the applied EF. (b) Probability density distribution of ϕ, the angle in the *y*–*z* plane between the projection of PC_1_ and the rotating CPEF vector.

We also calculated the angle ϕ. For the CPEF condition, ϕ is defined as the angle between the EF vector E⃗ and the projection of PC_1_ onto the *y*–*z* plane (PC_1_^⊥^), since E⃗ rotates within that plane. For the 0EF, SEF, and AEF conditions, where E⃗ is directed along the *x*-axis, ϕ is measured between the *y*-axis and PC_1_^⊥^. As shown in Figure 13b, ϕ follows a uniform distribution under 0EF, SEF, and AEF, indicating that the graphene aggregate’s orientation in the *y*–*z* plane is random and unaffected by the EF. In contrast, under CPEF, a peak appears at a value slightly less than + π/6, demonstrating that as the EF rotates in the *y*–*z* plane around the *x*-axis, the graphene aggregate rotates in sync but lags behind the field by an angle of approximately π/6.

#### 3.2.4 EF Impact Configurations of Aggregates

As shown in Figure 14, the state points of the aggregate configurations are plotted in the space of the top two principal moments (*p*_1_, *p*_2_), with each point colored according to the third principal moment (*p*_3_). In Figure 14a, under the 0EF condition, the data points cluster in the lower-left region where *p*_1_ is small, indicating a globular-like aggregate configuration. The inset in the panel illustrates the arrangement of individual graphene flakes within this aggregate. Unlike the arrangement observed for the smaller **G2**–**1** flakes (Figure 7a), the larger **G2**–**2** flakes align with their long axes oriented in the same direction.

**Figure 14:**
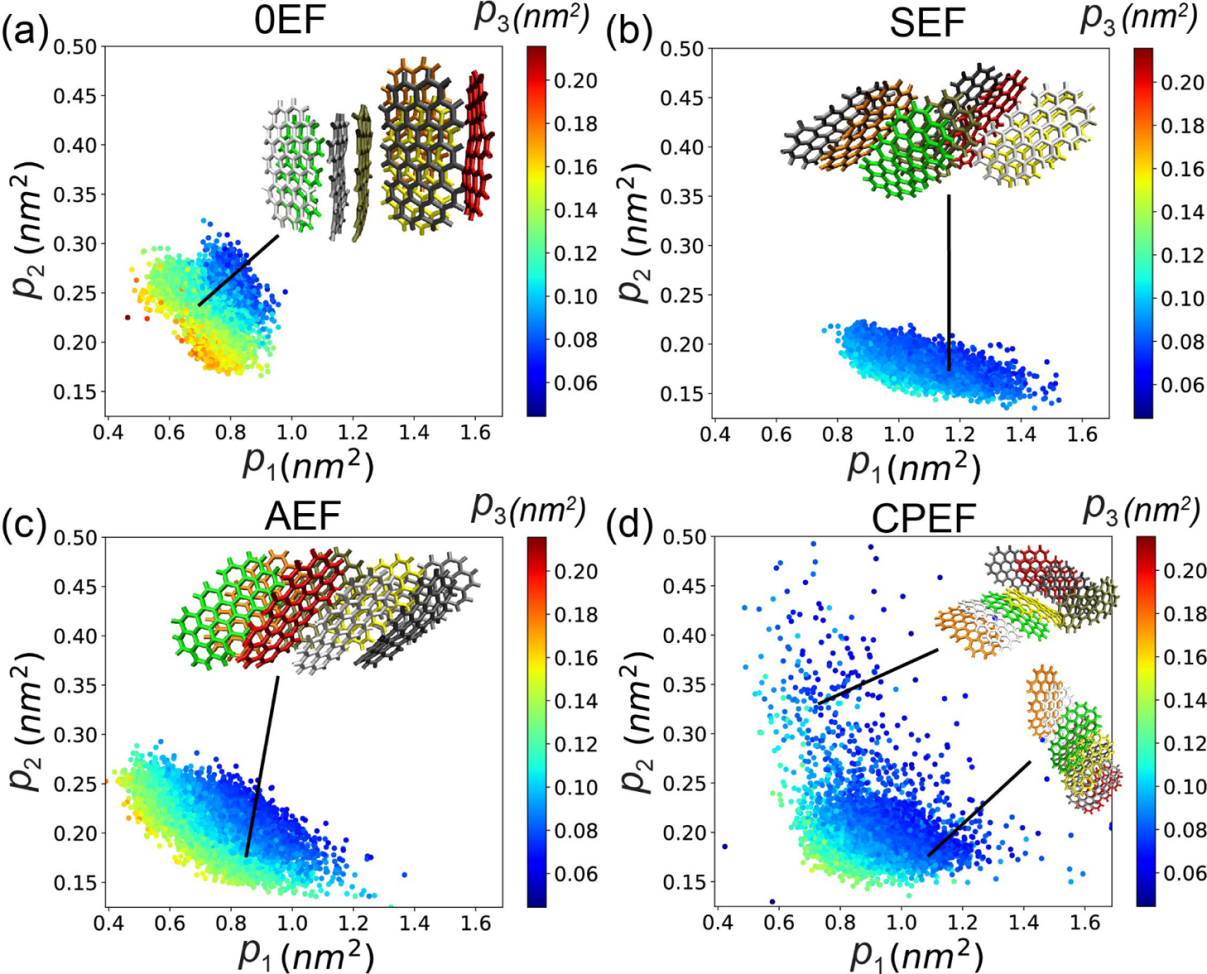
Distribution of **G2**–**2** aggregate configurations in the low-dimensional space defined by the principal moments (*p*_1_, *p*_2_) under the following conditions: (a) 0EF, (b) SEF, (c) AEF, and (d) CPEF. Each data point is colored according to the value of the third principal moment, *p*_3_.

When a directional EF (SEF or AEF) is applied, the data points spread toward regions with larger *p*_1_ values, indicating that the field stretches the aggregate configuration. As shown in the insets of panels b and c, this stretching is achieved by offsetting the graphene flakes relative to one another while maintaining parallel alignment of their long axes. Comparing panels b and c, we observe that under SEF, the data points exhibit slightly larger *p*_1_ and smaller *p*_2_ values, reflecting a thinner and longer aggregate configuration. In contrast, under AEF, the configuration is slightly shorter and wider.

Under CPEF, as shown in panel d of Figure 14, data points are distributed over a larger area, particularly extending into the upper region where *p*_2_ values are high. As illustrated in the inset of panel d, these configurations are significantly wider, exhibiting greater extension along the direction of the second principal component. In these structures, the long axes of the graphene flakes are no longer parallel, a result of the disruptive effect of the rotating CPEF, which breaks the ordered arrangement of flakes observed under the other three EF conditions.

For **G2**–**2** under CPEF, we did not observe the formation of smaller aggregate fractions as seen with the smaller **G2**–**1** flakes under the same condition. In Figure 14d, all data points represent aggregates of size 8. This occurs because the larger **G2**–**2** flakes exhibit stronger pairwise interactions, allowing the aggregate to remain intact as a single unit despite the disruptive influence of the CPEF, which disturbs the internally ordered structure. As shown in Figure S10, the binding energy between two **G2**–**2** graphene flakes is significantly stronger than that between **G2**–**1** graphene flakes.

The two-dimensional free energy landscape in the (*p*_1_, *p*_2_) space for **G2**–**2** under each EF condition is shown in Figure 15. Panel a corresponds to the 0EF condition, where three free energy minima are located in the left region of the plot, coinciding with the agglomeration of data points (Figure 14a). These minima represent distinct aggregate configurations, with a representative snapshot from the primary minimum provided as an inset. Although the other two minima correspond to configurations that differ slightly from the inset, they are structurally similar, as indicated by their proximity in the (*p*_1_, *p*_2_) space and the presence of connecting free energy pathways. This suggests that the graphene aggregate can transition between these configurations when the system is at equilibrium during the simulation.

**Figure 15:**
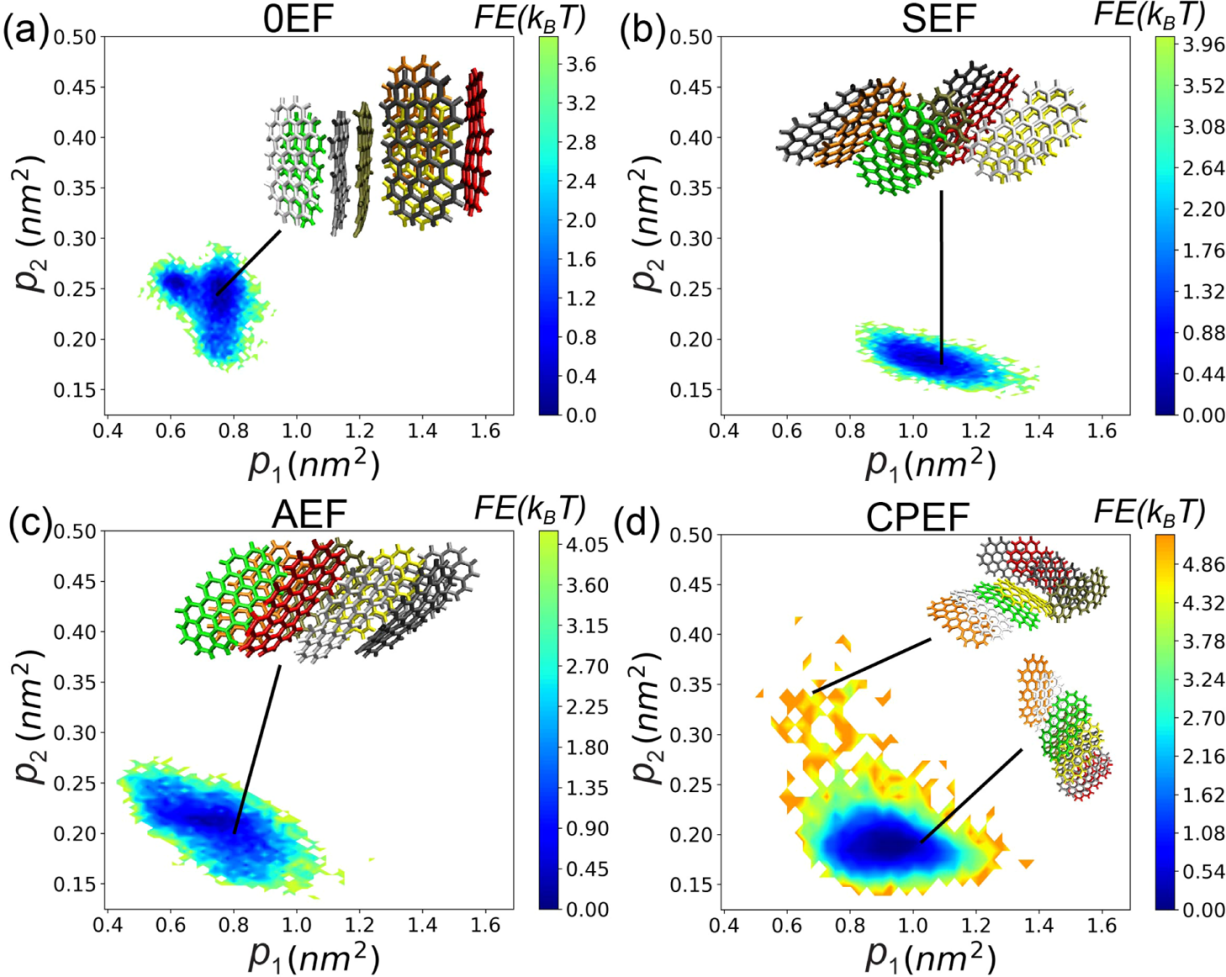
Two-dimensional free energy landscape of the **G2**–**2** aggregate in the principal moment space (*p*_1_, *p*_2_) for the following conditions: (a) 0EF, (b) SEF, (c) AEF, and (d) CPEF.

Under SEF and AEF, there are both single free energy minima located at the center of the point cloud, and typical aggregate configuration are shown as the insets in panel b and c of Figure 15, they are the most stable configuration during the simulation. For the CPEF condition, as the panel d shows, even though data points could spread over a large region of the plot (Figure 14d), the FE minima locates only at the lower region of the figure, where the configuration of the aggregate is elongated. For those “fat” configurations that have large *p*_2_ values and are located at the upper region, their corresponding FE is high, meaning that these configuration are not stable, they can be easily perturb by the CPEF, and changed to stable ones as the inset in the panel d shows.

## 4 Conclusions

In this work, molecular dynamics simulations are used to investigate the self-assembly behavior of graphene flakes under the influence of EF conditions. Two types of graphene flakes are studied: **G2**–**1**, which has a symmetric, round shape, and **G2**–**2**, which is larger and has a higher aspect ratio. Four electric field conditions are explored: 0EF, SEF, AEF, and CPEF. Various aggregation properties are quantitatively analyzed, including the number of bonds, average aggregate sizes, aggregate alignment and shape, and the free energy landscape in a low-dimensional space. More detailed conclusions are presented as follows:

Two types of metrics and criteria are used to characterize graphene–graphene binding: Metric A captures all types of intermolecular interactions, while Metric B is stricter and only identifies strong parallel-stacked configurations. For **G2**–**1**, more Metric A bonds are observed under the 0EF condition, and the application of an EF reduces the number of Metric A bonds. For **G2**–**2**, the EF has no significant impact on the number of Metric A bonds. The number of Metric B bonds remains largely unaffected by the EF for both **G2**–**1** and **G2**–**2** graphene aggregates.

Under 0EF, SEF, and AEF conditions, **G2**–**1** forms a single large aggregate as defined by Metric A, whereas CPEF disrupts these interactions, leading to smaller Metric A aggregates. In contrast, **G2**–**2** maintains a single aggregate under all EF conditions, a result of its larger size and stronger interflake interactions.

Under the 0EF condition, both **G2**–**1** and **G2**–**2** graphene aggregates adopt a globular, round-like configuration. The difference lies in the internal arrangement: graphene flakes are randomly oriented within the **G2**–**1** aggregate, whereas the long axes of the flakes are ordered in the **G2**–**2** aggregate. When an EF is applied, the aggregate becomes stretched and elongated, aligning with the direction of the field. This alignment results from the formation of 1D water nanostructures and directional hydrogen bonds under the EF; the elongated configuration minimizes disruption to this ordered network when aligned parallel to the field. Under CPEF, the stretched aggregate aligns within the *y*–*z* plane and rotates following the field, albeit with a characteristic lag angle.

The two-dimensional free energy landscape in the (*p*_1_, *p*_2_) space reveals the most stable aggregate configurations. When EFs are applied, the stable configuration of the graphene aggregate shifts from a globular, round-like shape to a stretched, rod-like structure. Under the CPEF condition, data points are distributed over the largest region, indicating that the aggregate explores a wider range of configurations compared to other EF conditions. Nevertheless, the 2D free energy landscape confirms that the most stable states under CPEF remain the stretched configurations.

This work deepens our understanding of how electric fields influence the self-assembly behavior of graphene flakes, potentially guiding future engineering applications for controlling graphene and other discotic materials. Future studies could focus on two key areas: (1) the impact of other polar organic solvents-such as *N*-methyl-2-pyrrolidone (NMP), dimethylformamide (DMF), dimethyl sulfoxide (DMSO), ethanol, and isopropyl alcohol (IPA)-on the self-assembly of graphene flakes under an electric field; and (2) the effect of external electric fields on DLC systems, particularly those featuring a rigid aromatic core with flexible peripheral side chains.

## Supplementary Material





## Notes

Supporting Information include: 1. Distribution of **G2**–**1** aggregate configurations in the low-dimensional space with each data point being colored according to the aggregate size. 2. Time evolution of the number of Metric A and Metric B bonds in the **G2**–**2** aggregate under different EF conditions. 3. Time evolution of the number of **G2**–**2** aggregates and their sizes. 4. Distribution of **G2**–**2** aggregate configurations in the (*p*_1_, *p*_2_) space, colored by the excluded volume (*V*_*ex*_). 5. The number of detected bonds as a function of the distance threshold. 6. Illustration of 1D hydrogen-bond network. 7. Properties of **G2**–**1** graphene under CPEF with 1.2 GHz frequency. 8. Illustration of graphene flakes in boxes with different sizes. 9. Calculation of the binding energy between two **G2**–**1** and **G2**–**2** graphene flakes. 10. Free energy landscapes of graphene flake aggregate obtained from the first and second half of the simulation trajectory. 11. Number of Metric A/B bonds for **G2**–**1** graphene from simulation trajectory using 2 and 1 fs time step (PDF)
Contains crucial input files for the simulation in Gromacs, including a modified gromos54a7 force field, topology and PDB/gro/itp files for **G2**–**1** and **G2**–**2** graphene flakes, itp file for spc water model. Atom mass, partial charges, and interaction coefficients can be found in these documents (ZIP)
