# Supplementary material for: Aggregation of Graphene Flakes Under Electric Field: A Molecular Simulation Study
Source: ACS Omega. 2026 Feb 3;11(6):10073–88. doi: 10.1021/acsomega.5c11004 (PMC12917640; doi:10.1021/acsomega.5c11004)
Supplement: Supplementary file 1 [file ao5c11004_si_001.pdf]

SUPPLEMENTARY MATERIAL

Aggregation of Graphene Flakes Under Electric Field: A Molecular Simulation Study

Jiang Wang,* Zaigui Yang, Yiping Shi, Guangxiang Wei, and Zhiling Li

College of Science, Guizhou Institute of Technology, Boshi Road, Huaxi District, Guiyang, Guizhou, 550025, China

E-mail: cwangjiang@git.edu.cn

SUPPLEMENTARY TEXT

In this section, we provide the proof that probability distribution function of θ to be $\frac{1}{2}\sin\theta$, and the probability distribution function of $[\cos\theta]$ to be $\frac{1}{2}$.

As shown in Figure S1, vector $\vec{r}$ is randomly pointed, the norm of $\vec{r}$ is R, only vectors in the narrow belt are within $[\theta, \theta + d\theta]$, and the area of the belt is $2\pi R^2 \sin\theta d\theta$, where R is the radius of the sphere, and the whole area of the sphere is $4\pi R^2$, so if $\vec{r}$ is uniformly distributed, the probability that $\vec{r}$ has its θ to be within $[\theta, \theta + d\theta]$ is the ratio of the belt area to the entire sphere area:

$$P(\theta \in [\theta, \theta + d\theta]) = \rho(\theta)d\theta = \frac{2\pi R^2 \sin\theta d\theta}{4\pi R^2} = \frac{1}{2}\sin\theta d\theta \tag{S1}$$

$$\Rightarrow \rho(\theta) = \frac{1}{2}\sin\theta \tag{S2}$$

Next, we would like to prove that if the vector $\vec{r}$ is randomly oriented, variable $[\cos\theta]$ as a constant probability distribution function (PDF) with the value being 0.5:

We first let $\cos\theta$ as a new variable y:

$$y = \cos\theta \tag{S3}$$

Since $\theta \in [0, \pi]$, so $y \in [-1, 1]$.

We have already shown that the probability distribution function (PDF) of θ is $\rho(\theta) = \frac{1}{2}\sin\theta$, from which, we can derive the cumulative distribution function (CDF) of θ as:

$$F_\theta(\theta) = P(0 < x < \theta) = \int_0^\theta \rho(x)dx = \int_0^\theta \frac{1}{2}\sin x dx = \frac{1}{2} - \frac{1}{2}\cos\theta \tag{S4}$$

For variable y, its CDF satisfies:

$$F_y(y_0) = P(-1 < y < y_0) = P(-1 < \cos\theta < y_0) = P(\pi > \theta > \arccos y_0) \tag{S5}$$

$$\because \theta \in [0, \pi] \tag{S6}$$

$$\therefore P(\pi > \theta > \arccos y_0) = 1 - P(0 < \theta < \arccos y_0) \tag{S7}$$

$$= 1 - F_\theta(\arccos y_0) \tag{S8}$$

$$= 1 - \left[\frac{1}{2} - \frac{1}{2}\cos\left(\arccos y_0\right)\right] \tag{S9}$$

$$= \frac{1}{2} + \frac{1}{2}y_0 \tag{S10}$$

$$\Rightarrow F_y(y) = \frac{1}{2} + \frac{1}{2}y \tag{S11}$$

$$\Rightarrow \rho(y) = \frac{dF_y(y)}{dy} = \frac{1}{2} \tag{S12}$$

This means that the variable [$\cos\theta$] has a constant value DPF, so it is uniformly distributed over [-1,1] with $\rho(\cos\theta) = \frac{1}{2}$.

SUPPLEMENTARY FIGURES

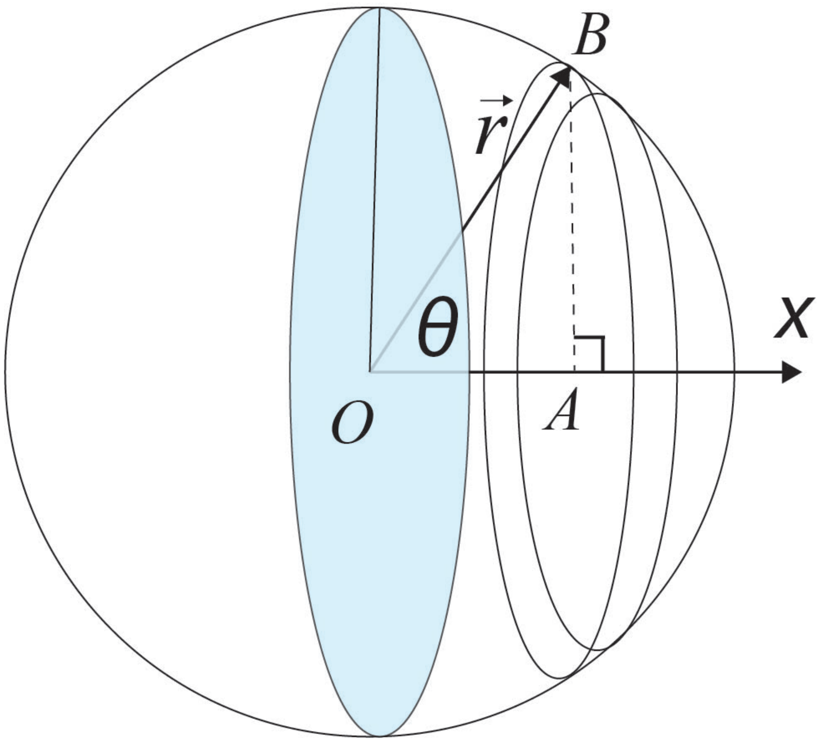

Figure S1: The illustration of the randomly oriented vector $\vec{r}$ and its corresponding angle θ.

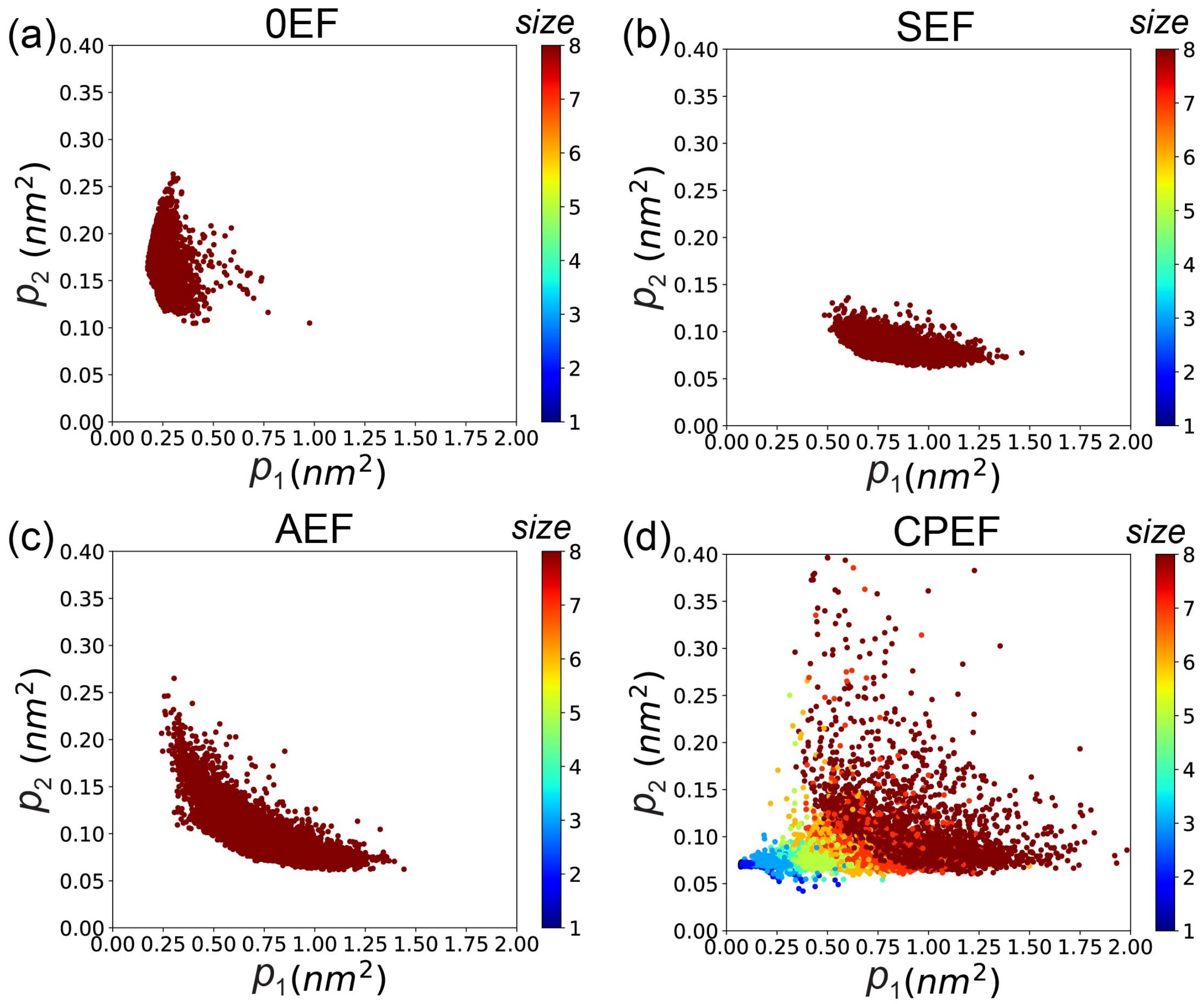

Figure S2: Distribution of **G2-1** aggregate configurations in the low-dimensional space defined by the principal moments (p_1, p_2) under the following conditions: (a) 0EF, (b) SEF, (c) AEF, and (d) CPEF. Each data point is colored according to the aggregate size.

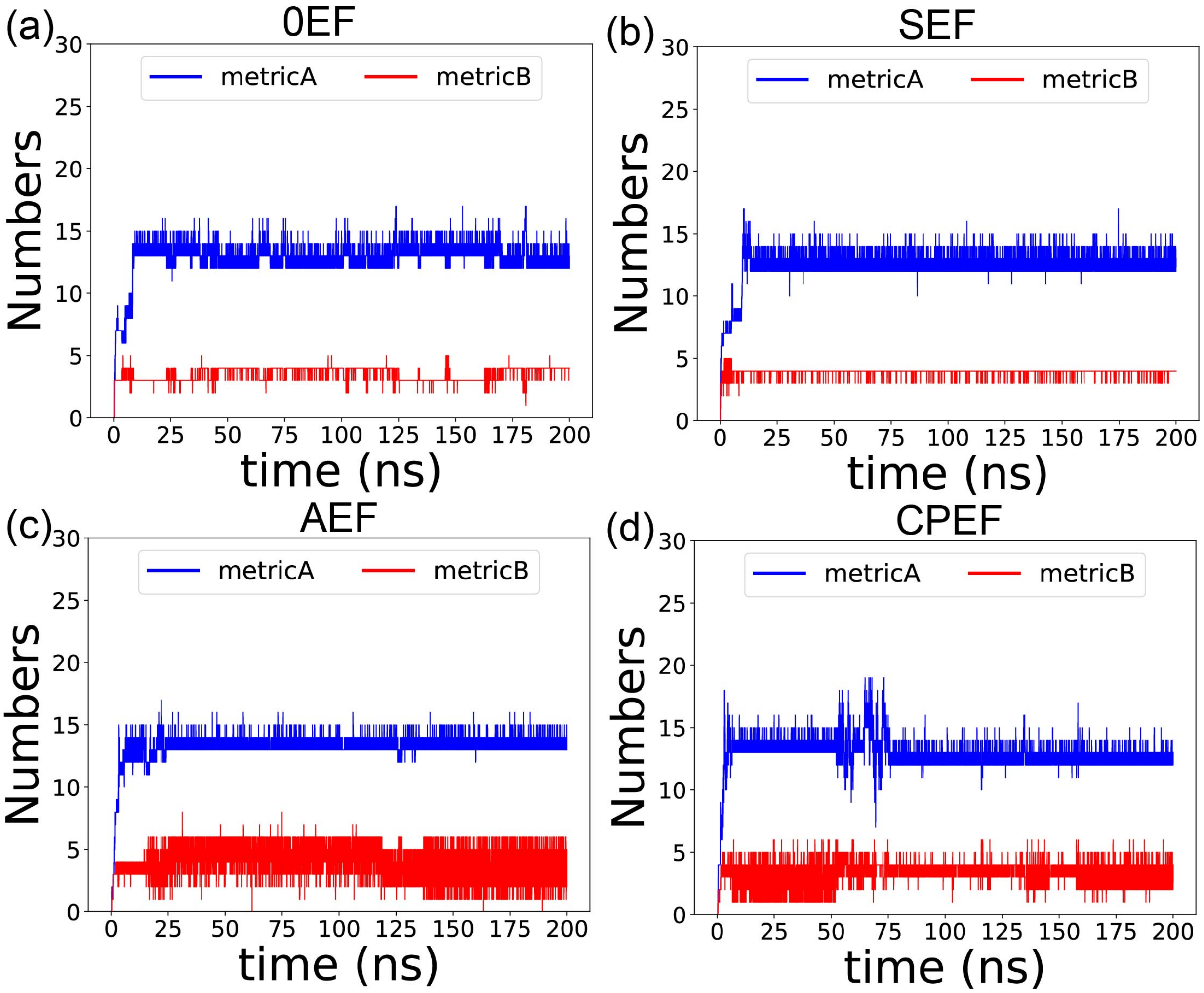

Figure S3: Time evolution of the number of Metric A and Metric B bonds in the **G2-2** aggregate under different EF conditions: (a) 0EF, (b) SEF, (c) AEF, and (d) CPEF.

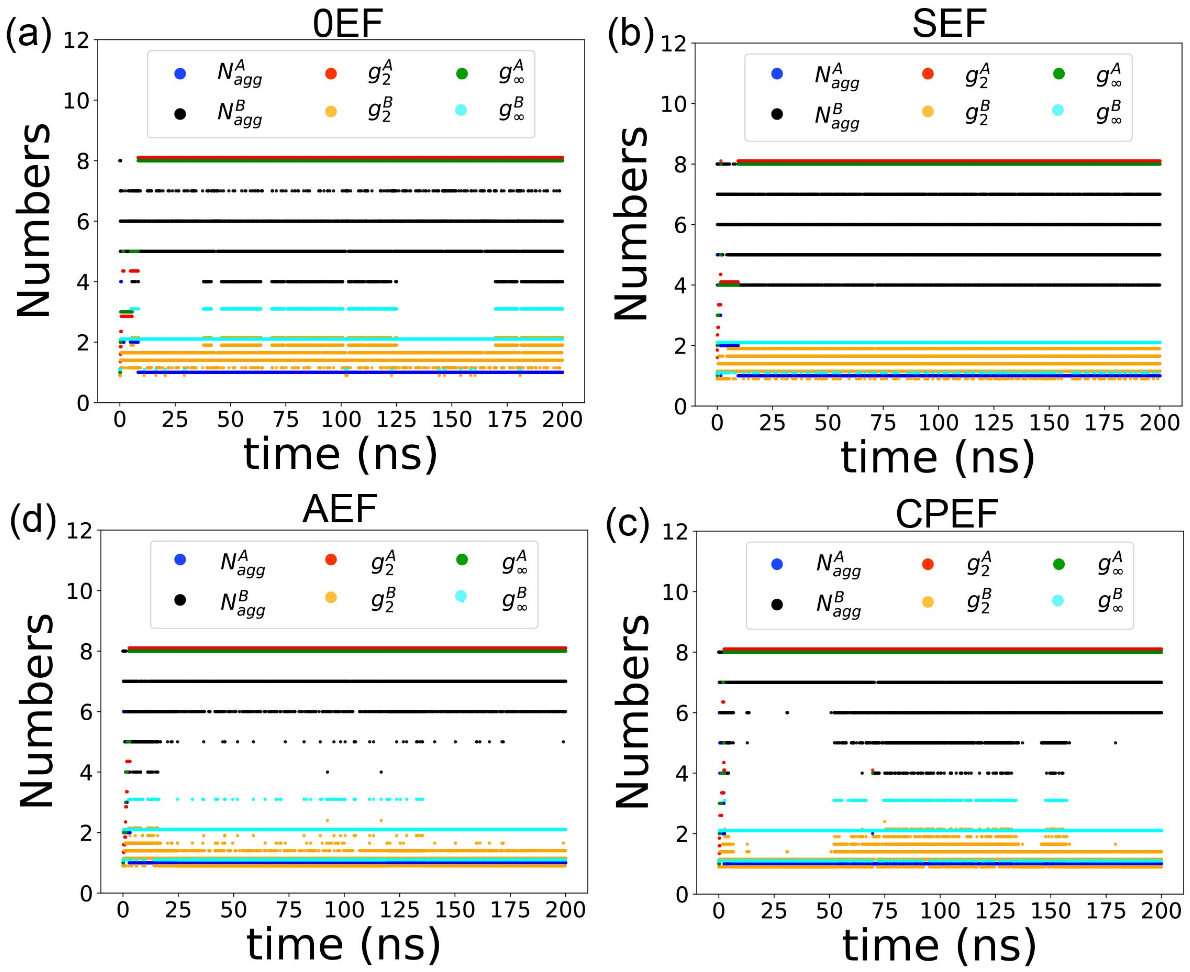

Figure S4: Time evolution of the number of **G2-2** aggregates and their sizes (based on Metric A/B) for the following conditions: (a) 0EF, (b) SEF, (c) AEF, and (d) CPEF.

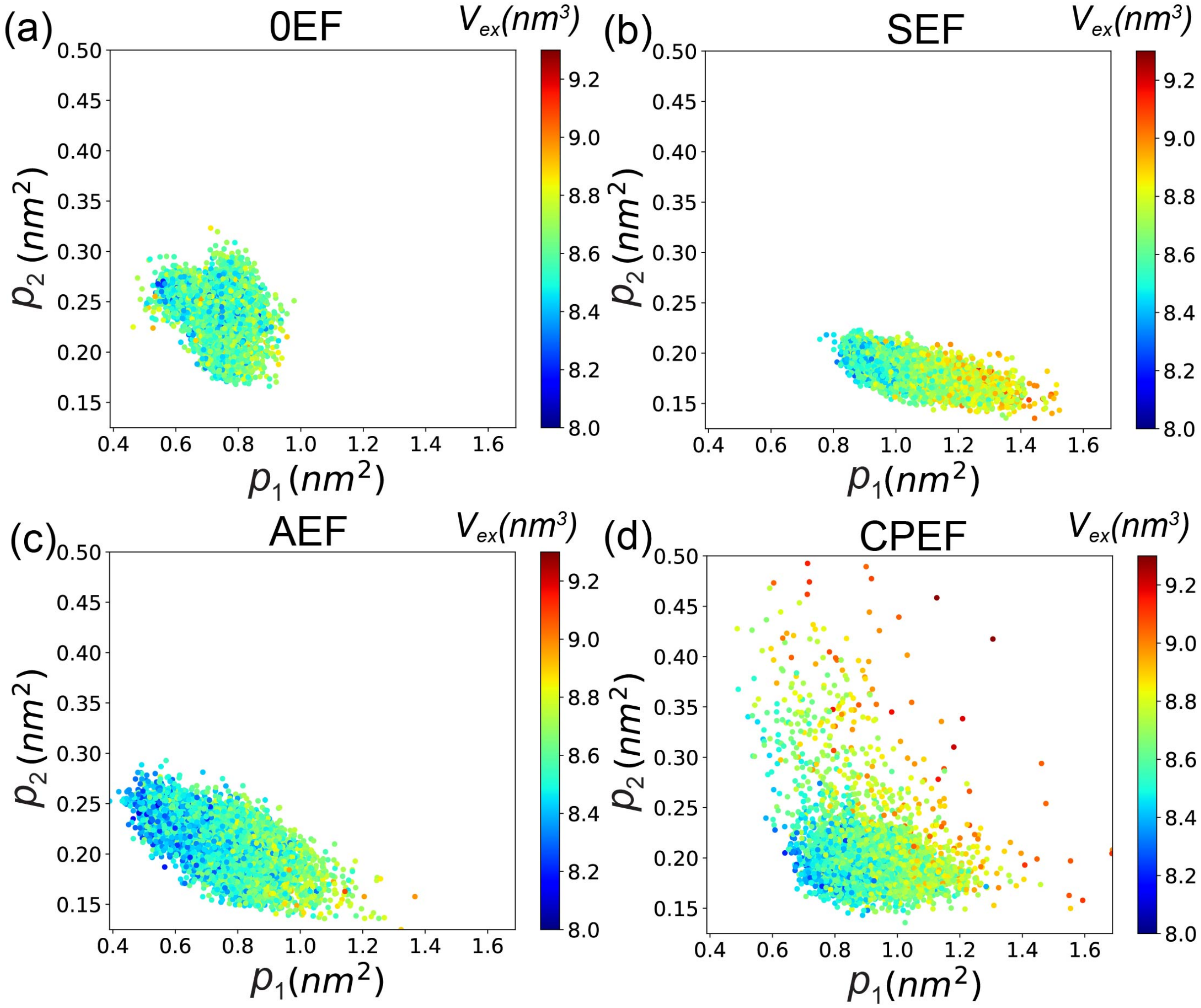

Figure S5: Distribution of **G2-2** aggregate configurations in the (p_1, p_2) space, colored by the excluded volume (V_{ex}), for the following conditions: (a) 0EF, (b) SEF, (c) AEF, and (d) CPEF.

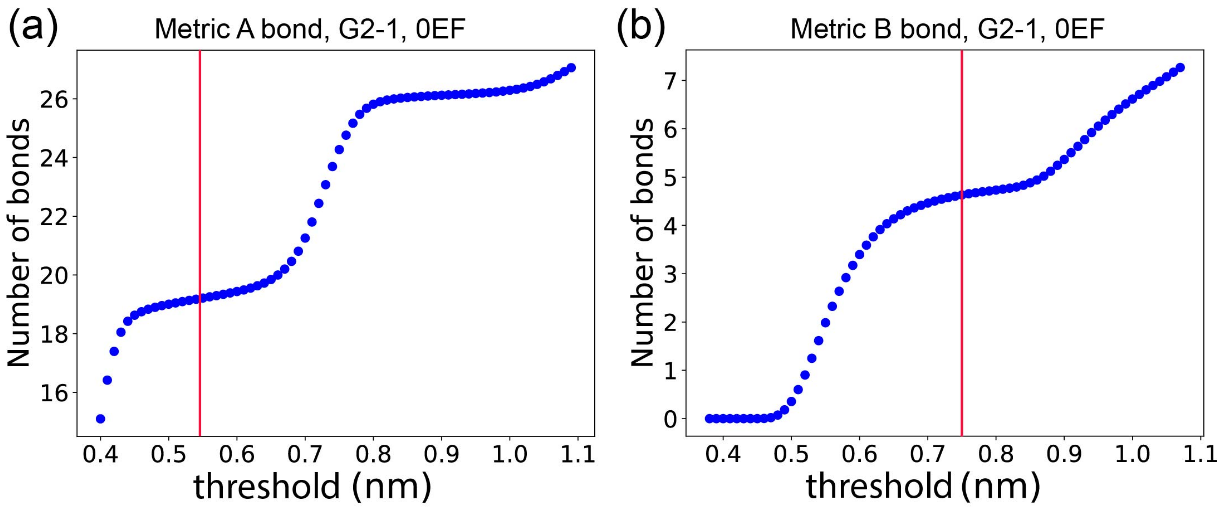

Figure S6: For the G2-1 graphene flake system under a zero electric field (0EF), the number of detected bonds is shown as a function of the distance threshold for (a) Metric A and (b) Metric B. Both plots exhibit a distinct plateau, where the bond count remains stable over a range of threshold values. The specific thresholds for Metric A and B were selected from within these flat regions, ensuring that bond detection is robust and insensitive to minor variations in the chosen cutoff distance.

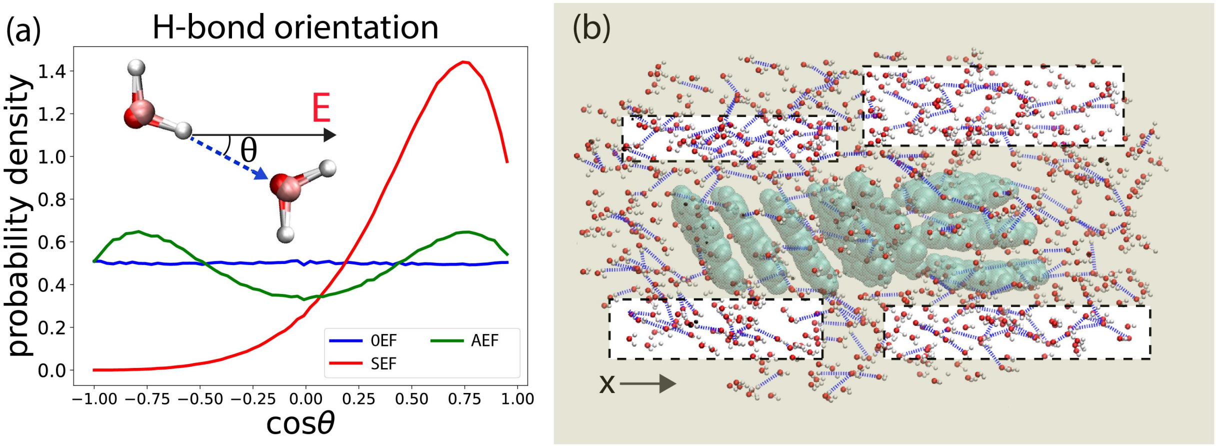

Figure S7: (a) Probability density distribution of H-bond orientation ($\cos\theta$) for water molecules under each EF condition. Hydrogen bond orientation for water molecule is pointing from H atom towards O atom. We can see that under 0EF, the H-bond are uniformly distributed, meaning that there is no directional tendency of H-bond driven by external EF, when SEF is applied, as the red curve shows, there is a significant peak located at $\cos\theta \approx 0.75$, where $\theta \approx \pi/4$, this means that all H-bond are impact by the SEF and have the tendency to be orient in the direction of EF, forming 1D structures, this 1D H-bond network could be clearly viewed in the highlighted region in panel (b). When AEF is applied, as EF is oscillating in the $\pm x$ direction, there are two peaks appear at the positive and negative region, meaning that water molecules are also flipping around with the oscillating AEF, so the orientation of the hydrogen bond change direction all the time. As to the CPEF, since the EF is rotating in the y-z plan, we could expect that there are also 1D hydrogen bond structures in y-z plan, and also following the rotating CPEF. (b) Snapshot showing 1D water clusters near the graphene flake aggregate; the snapshot was taken as the average of two adjacent frames; this could remove the noise and show out the most stable H-bonds.

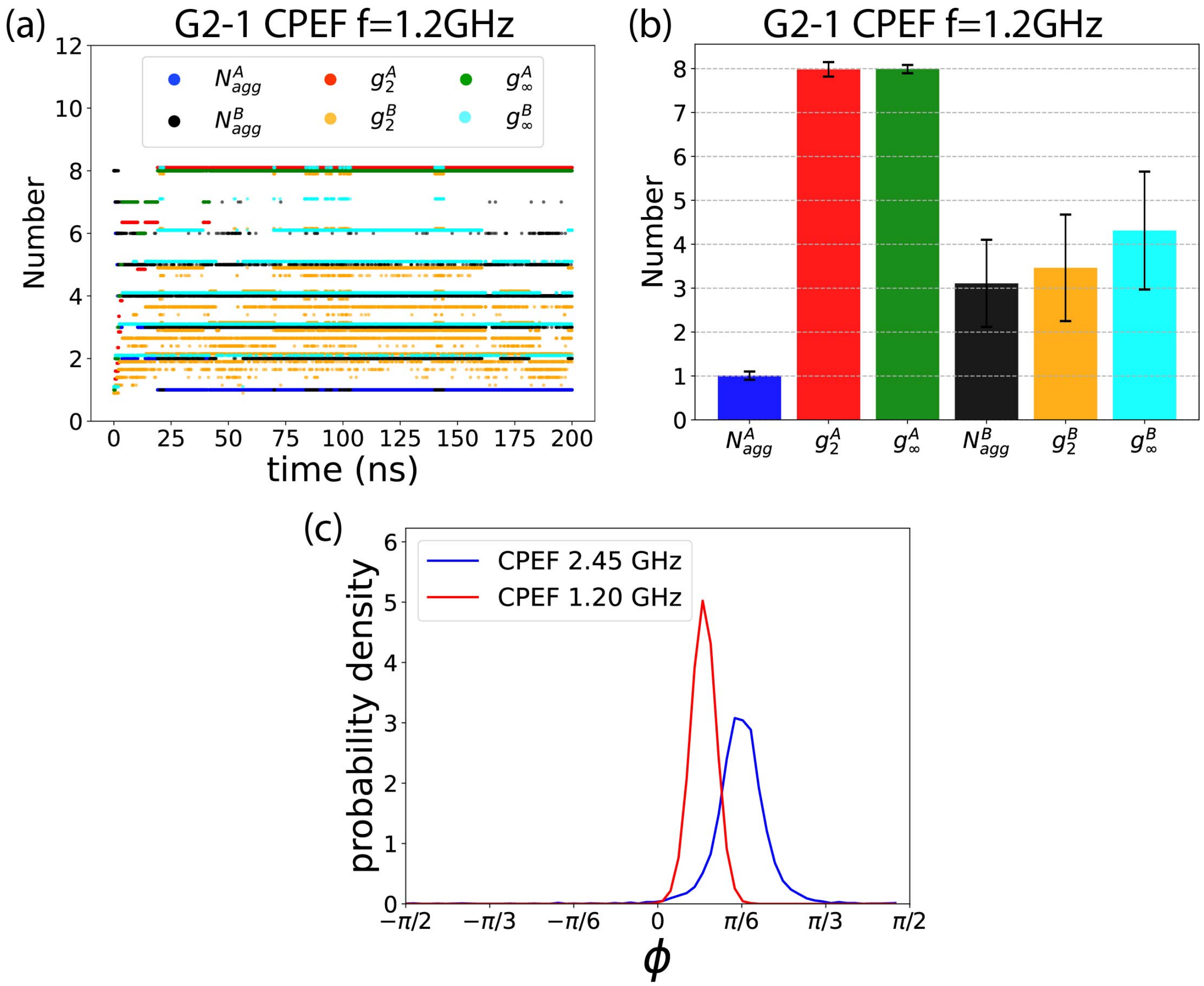

Figure S8: Simulation of G2-1 graphene under CPEF with 1.2 GHz frequency. (a) Time evolution of calculated aggregate number and sizes. (b) averaged aggregate numbers and sizes. (c) probability distribution of lag angle between EF and the aggregate.

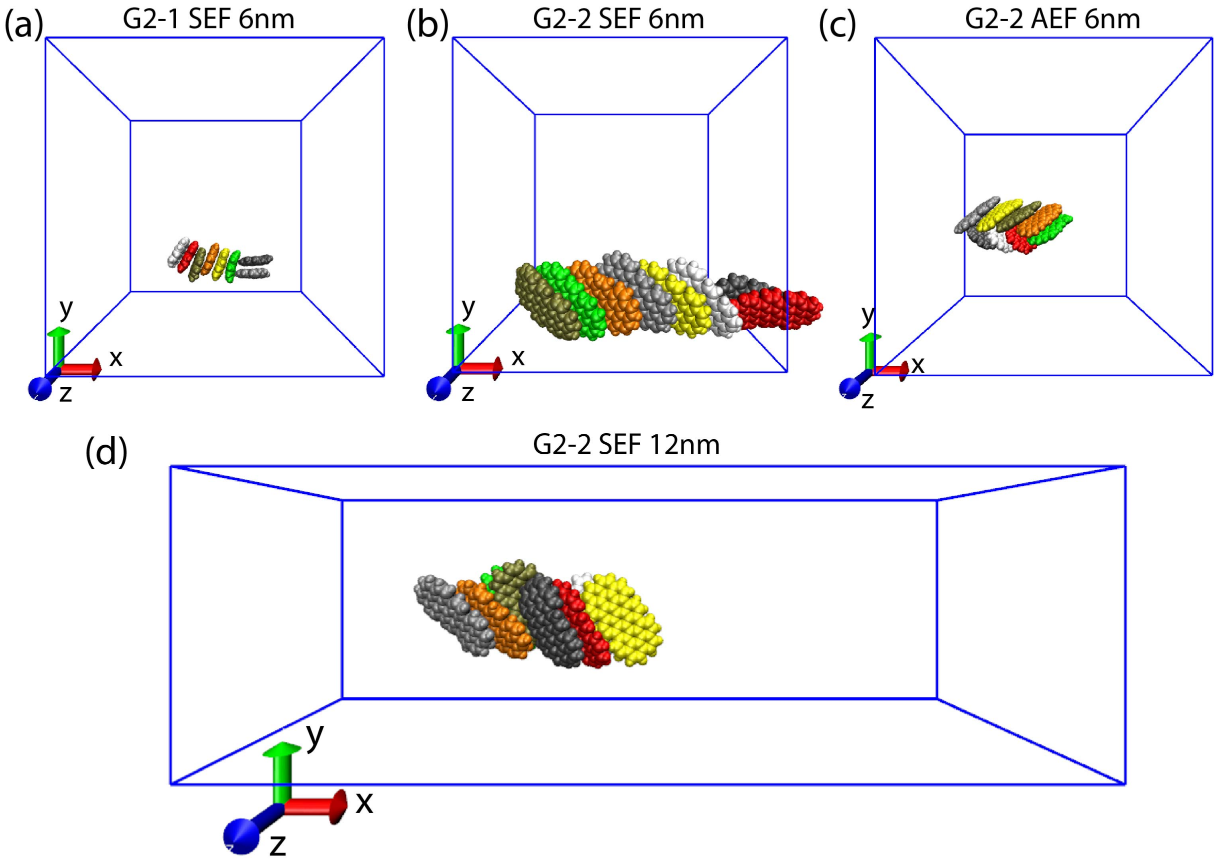

Figure S9: (a). G2-1 aggregate under SEF with the box size of 6nm. (b). G2-2 aggregate under SEF with the box size of 6nm, in this situation, graphene aggregate could interact with its own image. (c). G2-2 aggregate under AEF with the box size of 6nm. (d). G2-2 aggregate under SEF with the box length of 12nm.

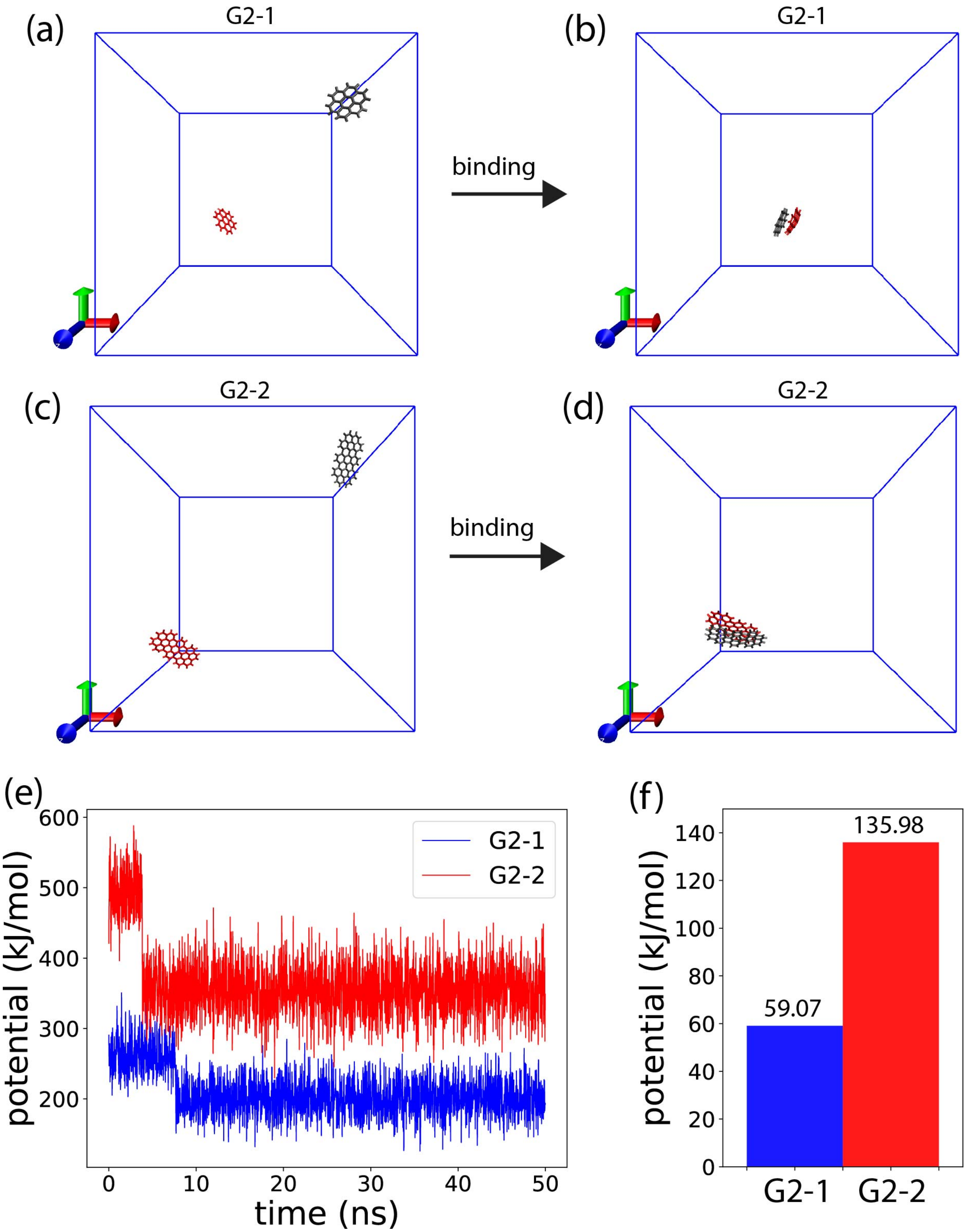

Figure S10: (a) Initial configuration of two G2-1 graphene flakes in water (water molecules are turned off for the sake of clarity). (b) G2-1 graphene flakes are bonded together. (c) Initial configuration of two G2-2 graphene flakes in water. (d) G2-2 graphene flakes are bonded together. (e) time evolution of the potential energy between G2-1 and G2-2 graphene flakes. (f) Calculated binding energy for G2-1 and G2-2.

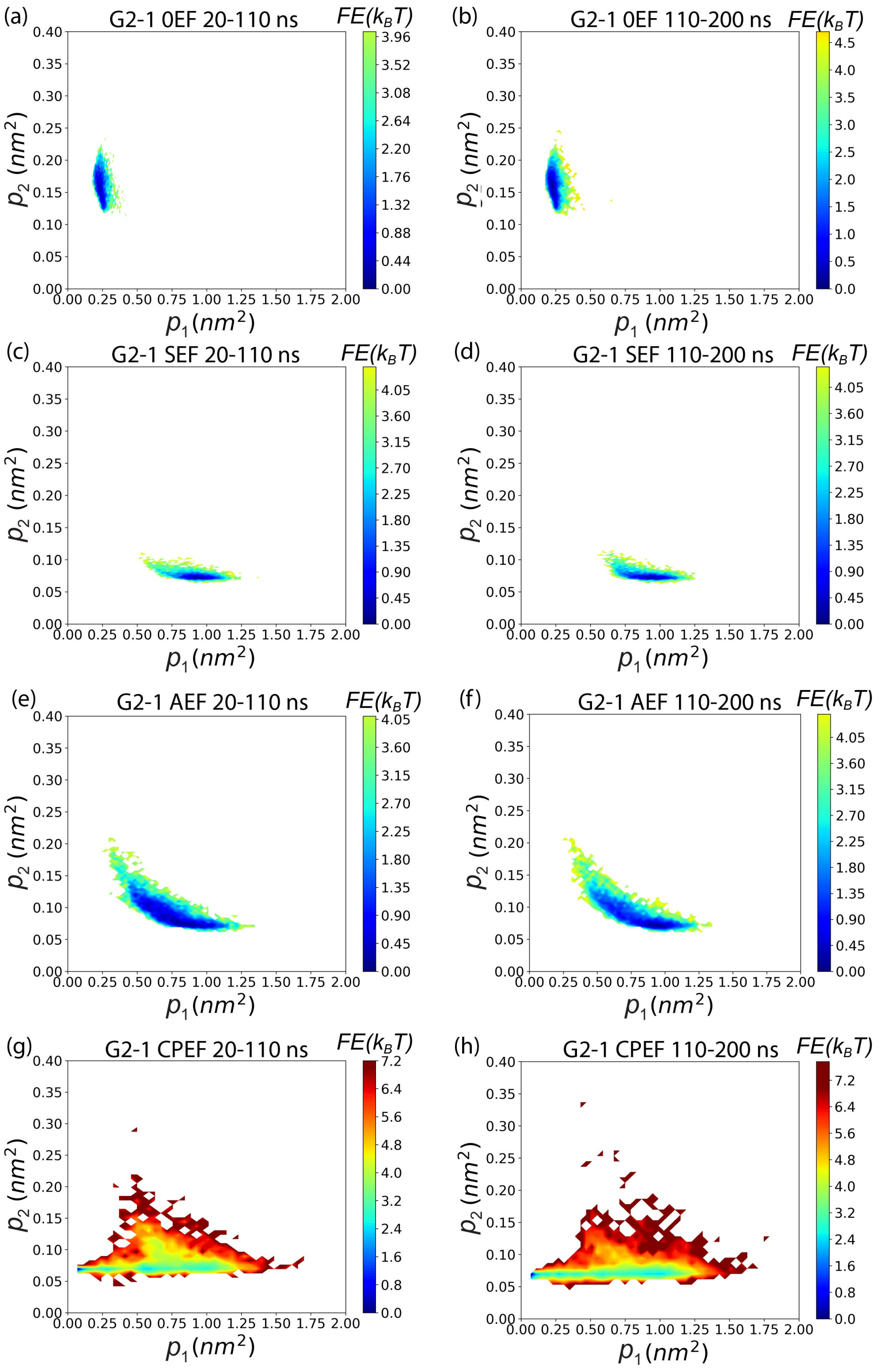

Figure S11: Free energy landscapes of graphene flake aggregate spanned by two principal components. Panel (a)(c)(e)(g) on the left column correspond to FE landscape obtained by using data points from 20-110 ns of the simulation trajectory. Panels (b)(d)(f)(h) on the right column are obtained using data from 110 – 200 ns of the simulation.

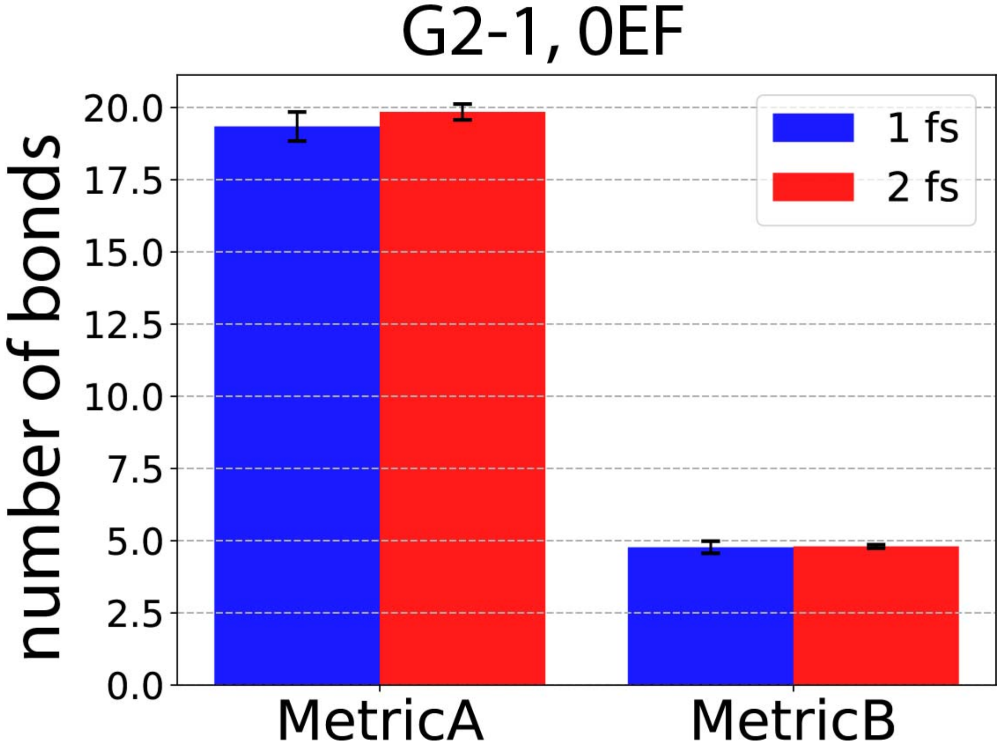

Figure S12: Number of Metric A/B bonds for G2-1 graphene flakes under 0EF condition, simulated using two timesteps, blue bars correspond to timestep = 1 fs, and red bars correspond to timestep = 2 fs.
